# Recent advances in the population biology and management of maize foliar fungal pathogens *Exserohilum turcicum*, *Cercospora zeina* and *Bipolaris maydis* in Africa

**DOI:** 10.3389/fpls.2024.1404483

**Published:** 2024-08-01

**Authors:** David L. Nsibo, Irene Barnes, Dave K. Berger

**Affiliations:** ^1^ Department of Plant and Soil Sciences, Forestry and Agricultural Biotechnology Institute (FABI), University of Pretoria, Pretoria, South Africa; ^2^ Department of Biochemistry, Genetics and Microbiology, Forestry and Agricultural Biotechnology Institute (FABI), University of Pretoria, Pretoria, South Africa

**Keywords:** Africa, maize, *Setosphaeria turcica*, *Cochliobolus heterostrophus*, population biology, northern corn leaf blight, grey leaf spot, turcicum leaf blight

## Abstract

Maize is the most widely cultivated and major security crop in sub-Saharan Africa. Three foliar diseases threaten maize production on the continent, namely northern leaf blight, gray leaf spot, and southern corn leaf blight. These are caused by the fungi *Exserohilum turcicum*, *Cercospora zeina*, and *Bipolaris maydis*, respectively. Yield losses of more than 10% can occur if these pathogens are diagnosed inaccurately or managed ineffectively. Here, we review recent advances in understanding the population biology and management of the three pathogens, which are present in Africa and thrive under similar environmental conditions during a single growing season. To effectively manage these pathogens, there is an increasing adoption of breeding for resistance at the small-scale level combined with cultural practices. Fungicide usage in African cropping systems is limited due to high costs and avoidance of chemical control. Currently, there is limited knowledge available on the population biology and genetics of these pathogens in Africa. The evolutionary potential of these pathogens to overcome host resistance has not been fully established. There is a need to conduct large-scale sampling of isolates to study their diversity and trace their migration patterns across the continent.

## 1 Introduction

Food demand driven by exponential human population growth over the past fifty years has shifted cropping systems from farms with high genotypic diversity to genetically uniform crops (termed monocultures) (Zhan et al., 2014). More recently, there has been an increased adoption of conservation agriculture (Rodenburg et al., 2020; Jat et al., 2021; Reicosky, 2021). These two factors have led to favorable conditions for crop pathogens that persist in the soil, including some foliar pathogens, to cause severe global disease outbreaks (Dill-Macky and Jones, 2000; Bateman et al., 2007; Simón et al., 2011; Bebber, 2015).

Maize production in the 2021/2022 production season was calculated at one billion tons (FAOSTAT, 2024). Yield has increased at a rate of 3.2% per year between 1972 and 2021 (Knoema, 2023). This is more than the 2.4% yield increase required per year to meet the expected global production demand by 2050 (Ray et al., 2013). Despite the cultural and food security importance of maize in many countries in sub-Saharan Africa, only 7.5% of the global maize crop is grown on the continent (FAOSTAT, 2024).

**Figure 1:**
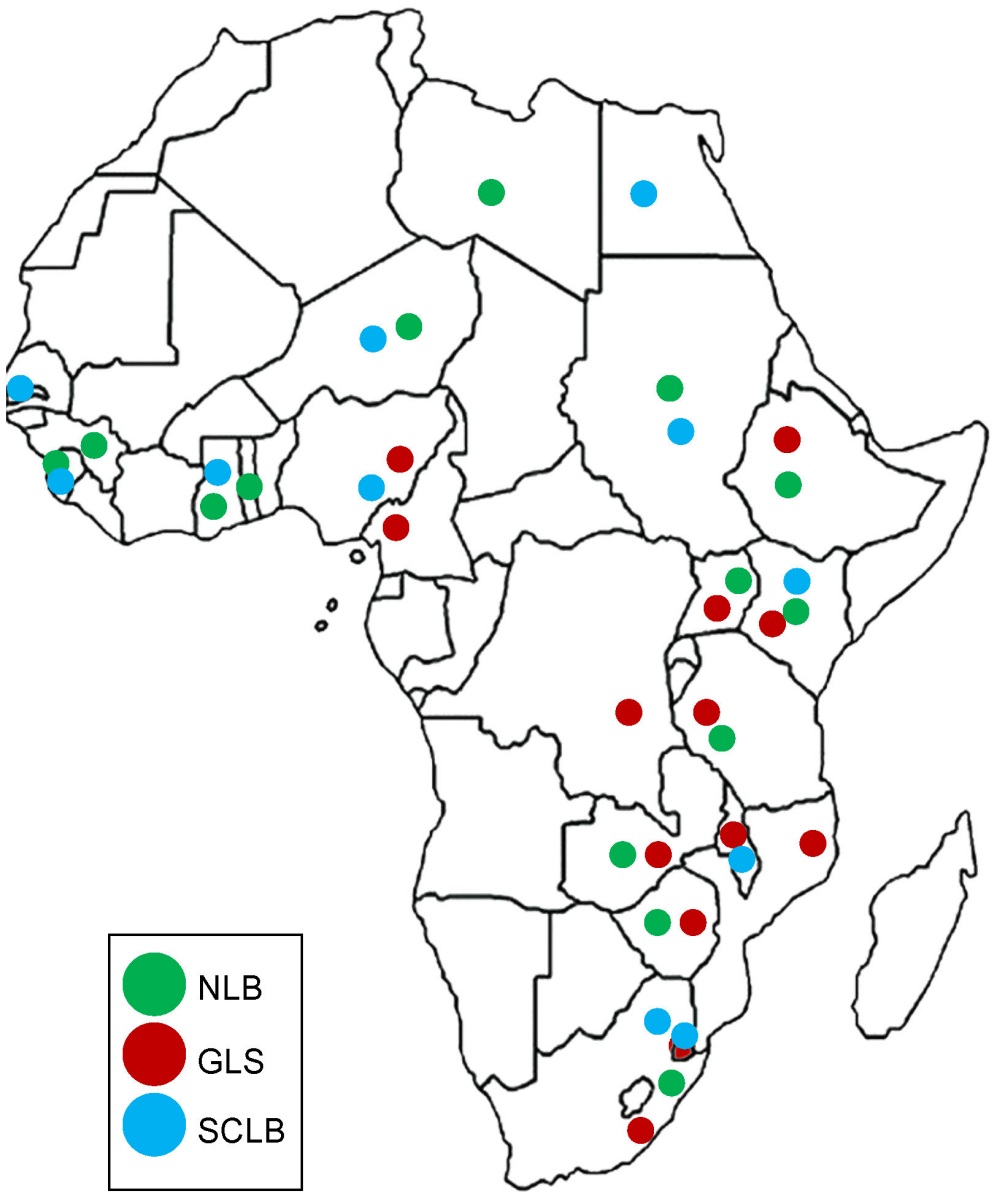
Distribution of three Maize leaf diseases in Africa. Northern leaf blight (NLB) is indicated in green, gray leaf spot (GLS) in red and southern corn leaf blight (SCLB) in blue. GLS is the most widely distributed disease in Africa, followed by NLB. Citations of the reports of the diseases in African countries are listed in **Table 1** and from the USDA database https://fungi.ars.usda.gov/.

A worldwide survey indicated that biotic factors were responsible for 23% of maize yield losses annually (Savary et al., 2019). The three foliar fungal diseases - northern (corn) leaf blight (NLB), gray leaf spot (GLS), and southern corn leaf blight (SCLB), contributed to more than 4% of these estimated yield losses (Savary et al., 2019). Sub-Saharan Africa yield losses for NLB were estimated at more than 1% (**Figure 1**). Unfortunately, survey data was not gathered for the other two diseases. However, GLS is widespread on the African continent (Nsibo et al., 2021), and SCLB has been reported from Egypt, Gambia, Ghana, Kenya, Malawi, Nigeria, Sierra Leone, South Africa, Sudan, and Eswatini (Rong and Baxter, 2006; Aregbesola et al., 2020) (**Figure 1**). Here, we review recent advances in the population biology and management of the causal pathogens of NLB, GLS, and SCLB, with a focus on Africa.

## 2 Global distribution and causal agents of NLB, GLS, and SCLB

### 2.1 Northern leaf blight

Northern leaf blight also known as Northern corn leaf blight (NCLB) or Turcicum leaf blight (TLB), has persisted for decades as a major foliar disease in maize producing regions of the World (Drechsler, 1923; Savary et al., 2019). Following its first discovery in Parma Italy, NLB was only well documented in the United States of America (USA) in 1878 and only emerged as an outbreak in 1889 (Drechsler, 1923). The disease has since appeared in the Americas, and Asia (Shi et al., 2017; Bashir et al., 2018; Mueller et al., 2020; Navarro et al., 2021). In Africa, NLB was first reported in Uganda in 1924 and has since been reported in several sub-Saharan African countries (**Figure 1**; **Table 1**) (Emechebe, 1975; Adipala et al., 1995; Abebe and Singburaudom, 2006; Haasbroek et al., 2014; Nieuwoudt et al., 2018; Berger et al., 2020).

**Table 1:** The epidemiological characteristics of NLB, GLS and SCLB causal pathogens.

	NLB	GLS	SCLB
**The first report**	1878, New Jersey, USA (Drechsler, 1923)	1925, Illinois, USA (Tehon and Daniels, 1925)	1923, USA (Drechsler, 1925; Robert, 1953)
**The first report in Africa**	1924, Uganda (Emechebe, 1975)	1988, KwaZulu-Natal South Africa (Ward et al., 1997)	1974, Mpumalanga, South Africa (Rong and Baxter, 2006)
**Causal pathogen (s) in Africa**	*Exserohilum turcicum* (Pass.) K.J. Leonard & Suggs	*Cercospora zeina* (Crous & U. Braun)	*Bipolaris maydis* (Y. Nisik. & C. Miyake) Shoemaker
**Old teleomorph name for genera**	*Setosphaeria* (Alcorn, 1988)	Unknown	*Cochliobolus* (Alcorn, 1988)
**Pathogen lifestyle**	Hemibiotroph (Ohm et al., 2012)	Necrotroph (Benson et al., 2015)	Necrotroph (Ohm et al., 2012)
**Lesion structure**	Long oblong, cigar-shaped, tan or grayish (White, 1999)	Rectangular, tan to grey lesions delimited within veins (Latterell and Rossi, 1983)	Spindle-shaped (Race T) and rectangular parallel sided lesions (Race O) with chlorotic borders (Jeffers, 2004)
**Lesion length**	2.5 to 30 cm (White, 1999)	0.5 to 7 cm (Latterell and Rossi, 1983)	2 to 3 cm (Jeffers, 2004)
**Asexual structures**	Pale to olivaceous brown straight or slightly curved conidia with a hilum, 5-10 septa (Alcorn, 1988).	70-180 × 2-3 µm hyaline, 6-10 septa (Crous et al., 2006)	Fusoid, straight or curved conidia with one germ tube from each end and protuberant conidial hilum, 2-8 septa (Alcorn, 1988)
**Sexual structure**	Pseudothecia reported *in-vitro* (Abadi et al., 1993)	Unknown	Ascospores reported when intercrossed with other *Helminthosporium* spp. (Nelson, 1960).
**Optimal growth conditions**	18-27°C and high humidity	Temperature 22-30°C, relative humidity is > 90%	20-32°C and high humidity
**Toxin production in culture**	Monocerin (Robeson and Strobel, 1982)	Cercosporin (*C. zeae-maydis*) (Wang et al., 1998). Unknown toxin for *C. zeina*	Race T produces toxins I, II, III and IV (Karr et al., 1974)

The causal pathogen of NLB is *Exserohilum turcicum* (Pass.) K.J. Leonard & Suggs, which is the asexual form of this hemibiotrophic Dothideomycete (Leonard and Suggs, 1974). Researchers have defined physiological races of *E. turcicum* based on six maize qualitative resistance genes namely; *Ht1, Ht2, Ht3, Htn1, Htn2*, and *Htm1*, that *E. turcicum* is able to overcome (Jordan et al., 1983; Jindal et al., 2019; Navarro et al., 2021; Muñoz-Zavala et al., 2023). The race groups are determined based on the screening of a differential set of maize lines, each with a different resistance gene (Leonard et al., 1989). Routine screening of *E. turcicum* races is carried out using maize differential genotypes in some maize producing countries (Weems and Bradley, 2018; Jindal et al., 2019; Turgay et al., 2020; Navarro et al., 2021; Muñoz-Zavala et al., 2023). However, maize resistance responses in these germplasm sets are highly variable and dependent on growth room/glasshouse conditions, and genetic background effects (Weems and Bradley, 2018; Jindal et al., 2019). To date, there are 29 described race groups based on the combinations of maize resistance genes that can be overcome, with race 0 defined as being able to overcome all known resistance genes (**Table 2** – see references within). Race 0 has been described from countries in all the continents except South America (**Table 2**). Twelve of the race combinations have been reported from African countries (**Table 2**), although screening has only been done on *E. turcicum* isolates from South Africa, Kenya, Uganda, and Zambia (Craven and Fourie, 2011).

**Table 2:** The physiological races of *Exserohilum turcicum* and *Bipolaris maydis* and their global distribution.

Disease	Race	Resistance in host that is overcome by the Race	Distribution by continent and country					Authors references
			Africa	Asia	Europe	North America	South America	
**Northern leaf blight**	0	Virulent to all known R genes	Kenya, South Africa, Uganda, Zambia	China	Germany	Canada, USA		Sun et al., 2005; Dong et al., 2008; Zhao et al., 2008; Muiru et al., 2010; Craven and Fourie, 2011; Ramathani et al., 2011; Weems and Bradley, 2018; Jindal et al., 2019
	1	Ht1	Kenya	China, Israel	Austria, Germany, Hungary	Canada, USA	Brazil	Abadi et al., 1989; Sun et al., 2005; Ferguson and Carson, 2007; Dong et al., 2008; Muiru et al., 2010; Ramathani et al., 2011; Weems and Bradley, 2018; Jindal et al., 2019
	2	Ht2	Kenya, Uganda	China	Austria, Germany	Canada, USA	Brazil	Jordan et al., 1983; Sun et al., 2005; Zhao et al., 2008; Muiru et al., 2010; Ramathani et al., 2011; Weems and Bradley, 2018; Jindal et al., 2019
	3	Ht3	Kenya	China	France, Italy	Canada, USA		Sun et al., 2005; Dong et al., 2008; Zhao et al., 2008; Muiru et al., 2010; Ramathani et al., 2011; Jindal et al., 2019
	12	Ht1, Ht2	Kenya	China	Germany	Canada, USA		Sun et al., 2005; Dong et al., 2008; Zhao et al., 2008; Muiru et al., 2010; Weems and Bradley, 2018; Jindal et al., 2019
	13	Ht1, Ht3	Kenya, Zambia	China	Germany	Canada, USA	Mexico	Dong et al., 2008; Zhao et al., 2008; Muiru et al., 2010; Zhu et al., 2011; Weems and Bradley, 2018
	23	Ht2, Ht3	Kenya, Zambia	China	Germany	Canada, USA	Mexico	Sun et al., 2005; Ferguson and Carson, 2007; Dong et al., 2008; Muiru et al., 2010; Zhu et al., 2011
	123	Ht1, Ht2, Ht3	Kenya	China		USA		Sun et al., 2005; Zhao et al., 2008; Muiru et al., 2010; Weems and Bradley, 2018
	N	Htn1	Kenya, Uganda	China		Canada		Sun et al., 2005; Dong et al., 2008; Muiru et al., 2010; Weems and Bradley, 2018; Jindal et al., 2019
	1N	Ht1, Htn1		China		Canada, USA	Brazil	Gianasi et al., 1996; Dong et al., 2008; Weems and Bradley, 2018; Jindal et al., 2019
	2N	Htn2, Htn1		China		USA	Mexico	Windes and Pederson, 1991; Welz et al., 1993; Dong et al., 2008
	3N	Ht3, Htn1	Kenya	China	France, Italy	Canada		Windes and Pederson, 1991; Welz et al., 1993; Dong et al., 2008
	12N	Ht1, Ht2, Htn1		China		Canada	Brazil	Gianasi et al., 1996; Dong et al., 2008; Jindal et al., 2019
	13N	Ht1, Ht3, Htn1	Kenya, South Africa	China		Canada		Sun et al., 2005; Muiru et al., 2010; Human et al., 2020
	23N	Ht2, Ht3, Htn1	Kenya, Uganda, South Africa, Zambia	China	Austria, Germany	USA	Mexico	Sun et al., 2005; Ferguson and Carson, 2007; Dong et al., 2008; Zhao et al., 2008; Muiru et al., 2010; Human et al., 2020
	123N	Ht1, Ht2, Ht3, Htn1		China			Brazil	Gianasi et al., 1996; Dong et al., 2008; Ma et al., 2020
	M	Htm1				Canada, USA		Weems and Bradley, 2018; Jindal et al., 2019
	1M	Ht1, Htm1				Canada, USA		Weems and Bradley, 2018; Jindal et al., 2019
	2M	Ht2, Htm1				Canada		Zhu et al., 2011; Jindal et al., 2019
	13M	Ht1, Ht3, Htm1				Canada		Inderbitzin et al., 2010; Jindal et al., 2019
	3M	Ht3, Htm1				Canada		Hulme, 2009; Jindal et al., 2019
	23M	Ht2, Ht3, Htm1				USA		Weems and Bradley, 2018; Jindal et al., 2019
	MN	Htm1, Htn1				USA		Weems and Bradley, 2018
	1MN	Ht1, Htm1, Htn1				Canada, USA		Weems and Bradley, 2018; Jindal et al., 2019
	2MN	Ht2, Htm1, Htn1				USA		Weems and Bradley, 2018
	12MN	Ht1, Ht2, Htm1, Htn1				Canada, USA		Zhu et al., 2011; Weems and Bradley, 2018; Jindal et al., 2019
	13MN	Ht1, Ht3, Htm1, Htn1				Canada		Jindal et al., 2019
	23MN	Ht2, Ht3, Htm1, Htn1				USA		Weems and Bradley, 2018; Jindal et al., 2019
	123MN	Ht1, Ht2, Ht3, Htm1, Htn1				Canada		Jindal et al., 2019
**Southern corn leaf blight**	0	Virulent for all	All maize producing countries	All maize producing countries	All maize producing countries	All maize producing countries	All maize producing countries	Smith et al., 1970; Mwangi, 1998; Balint-Kurti et al., 2007; Wang et al., 2010; Pavan and Shete, 2021
	T	cms-T	South Africa			USA		Leonard, 1977a, b; Rong and Baxter, 2006; Wang et al., 2010
	C	cms-C		China				Wei et al., 1988; Gao et al., 2000
	S	cms-S		China				Wang et al., 2010; Zhao et al., 2012

Research on maize resistance genes to *E. turcicum* has revealed that the *Htn1* gene encodes ZmWAK-RLK1, a wall-associated receptor-like kinase (Hurni et al., 2015). Further research provided evidence that maize *Ht2* and *Ht3* genes encoded the same ZmWAK-RLK which corresponded to a different allele of ZmWAK-RLK1 (Yang et al., 2021). This is consistent with previous work that *Htn1*, *Ht2*, and *Ht3* map to chromosome 8 (Yang et al., 2021). This brings into question the validity of using *Ht* genes only to allocate *E. turcicum* race classes when screening with differential maize panels. The authors propose that responses may be confounded by “modifier genes” as a result of (i) different genetic backgrounds, and (ii) variation in the size of the introgression surrounding an *Ht* gene in each differential maize line (Yang et al., 2021). This may explain why some isolates of *E. turcicum* gave different responses and were thus classified as race 2 or race 3 on the “differential” maize lines carrying *Ht*2 and *Ht*3 genes. In addition, it is well known amongst maize pathologists that responses to the pathogen are very sensitive to environmental conditions, which confounds reproducibility in *E. turcicum* race screening using differential panels (Weems and Bradley, 2018).

### 2.2 Gray leaf spot

Globally, GLS is the second most economically important foliar disease of maize after NLB, and is the most important foliar disease in the USA and Canada (Mueller et al., 2016, Mueller et al., 2020). Gray leaf spot disease was first reported in 1925 (Tehon and Daniels, 1925), and only became economically important in the late 1970s in the USA (Latterell and Rossi, 1983) and has since been reported in the Americas (Zhu et al., 2002; Juliatti et al., 2009; Neves et al., 2015; Mueller et al., 2020) and Asia (Manandhar et al., 2011; Liu and Xu, 2013). In Africa, GLS was first reported in 1988 in South Africa (Ward et al., 1997) and has since been reported in sub-Saharan Africa (**Figure 1**; **Table 1**).

*Cercospora zeae-maydis* Tehon & E.Y Daniels (Tehon and Daniels, 1925), and *Cercospora zeina* Crous & U. Braun (Crous and Braun, 2003) cause GLS. *Cercospora zeae-maydis* is predominant in the Americas and Asia, whereas *C. zeina* is found in Africa, Brazil, some parts of Asia, and the Eastern corn belt of the USA (Wang et al., 1998; Goodwin et al., 2001; Okori et al., 2003; Meisel et al., 2009; Neves et al., 2015; Duan et al., 2022). Although other *Cercospora* spp. have been associated with GLS, namely *Cercospora* spp. CPC 12062, a single isolate from South Africa (Crous et al., 2006) and *Cercospora sorghi* var. *maydis* Ellis & Everh. reported in Kenya (Kinyua et al., 2010) and Brazil (Neves et al., 2015), their role in pathogenicity has not been determined. The rest of this review will, therefore, focus on *C. zeina*, which is the predominant pathogen in Africa.

### 2.3 Southern corn leaf blight

Southern corn leaf blight, also known as Maydis leaf blight, was first reported in the USA in 1923 (Drechsler, 1925) and became a serious concern in the 1970s (Smith et al., 1970). Since then, SCLB reports have emerged from Western Europe, Asia and Africa (Munjal and Kapoor, 1960; Ullstrup, 1972; Fisher et al., 1976; Gregory et al., 1979; Carson et al., 2004; Singh and Srivastava, 2012; Manzar et al., 2022). The first report of SCLB in Africa was an outbreak in 1974 in South Africa, which resulted in the withdrawal of Texas male-sterile cytoplasm (T-cms) maize germplasm from the country’s breeding programs (Levings, 1990; Rong and Baxter, 2006) (**Table 1**). Since then, no reports on SCLB have emerged from the country and on the rest of the continent until two decades later in Kenya (Mwangi, 1998) and recently on seeds in Nigeria (Biemond et al., 2013), thus indicating that SCLB can be a seedborne disease. These reports indicate that SCLB is present in Africa (**Figure 1**), currently at levels where its severity and occurrence are still insignificant. However there is the potential of SCLB becoming a severe and serious phytosanitary threat to maize production in Africa.

*Bipolaris maydis* (Y. Nisik. & C. Miyake) Shoemaker is the causative pathogen of SCLB (Smith et al., 1970). Previously known as *Cochliobolus heterostrophus*, *B. maydis* has been adopted as the most widely accepted species name (Rossman et al., 2013). Four physiological races of *B. maydis* (races O, T, C, and S) are known globally (Smith et al., 1970; Wei et al., 1988; Levings, 1993; Manamgoda et al., 2014), while races C and S only exist in China (Wei et al., 1988; Wang et al., 2010; Zhao et al., 2012; Bruns, 2017) (**Table 2**).

Overall, the USA was the first to report NLB, GLS, and SCLB in the early 20th-century (Drechsler, 1925; Tehon and Daniels, 1925). This could have been a result of the USA’ system of Land Grant Universities with farmer extension services, vigilant crop disease diagnosis activities, and adoption of hybrid maize breeding (Duvick, 2001). At that time, maize production expanded and the planting of monocultures increased, creating a high risk of disease if susceptible genotypes were planted (Dodd, 2000; Duvick, 2001).

Gray leaf spot differs from NLB and SCLB, with no known physiological races among its populations. Physiological races of *E. turcicum* and *B. maydis* follow the “gene for gene” hypothesis. Races are classified based on a pathogen’s ability to overcome resistance genes in maize inbred lines, often by loss of an avirulence gene recognized by a specific maize resistance gene (Leonard et al., 1989). However, the GLS-maize pathosystem has no known virulence genes or major resistance genes, so there are no known physiological races.

## 3 Disease epidemiology and economic impact of NLB, GLS and SCLB on maize

Disease epidemiology entails an understanding of the dynamics of disease development and proliferation in space and time (Milgroom and Peever, 2003). Several biotic, abiotic, and edaphic factors contribute to plant diseases (Milgroom and Peever, 2003). The knowledge on the above mentioned predisposing factors and epidemiological parameters, such as infection efficiency, latent period, and spore production in disease development, is therefore crucial in deciding the nature of control strategies to adopt (de Vallavieille-Pope et al., 2000; Milgroom and Peever, 2003). This section reviews the epidemiology of NLB, GLS, and SCLB, the factors that favor their development, and their economic impact.

### 3.1 Northern leaf blight

**Figure 2:**
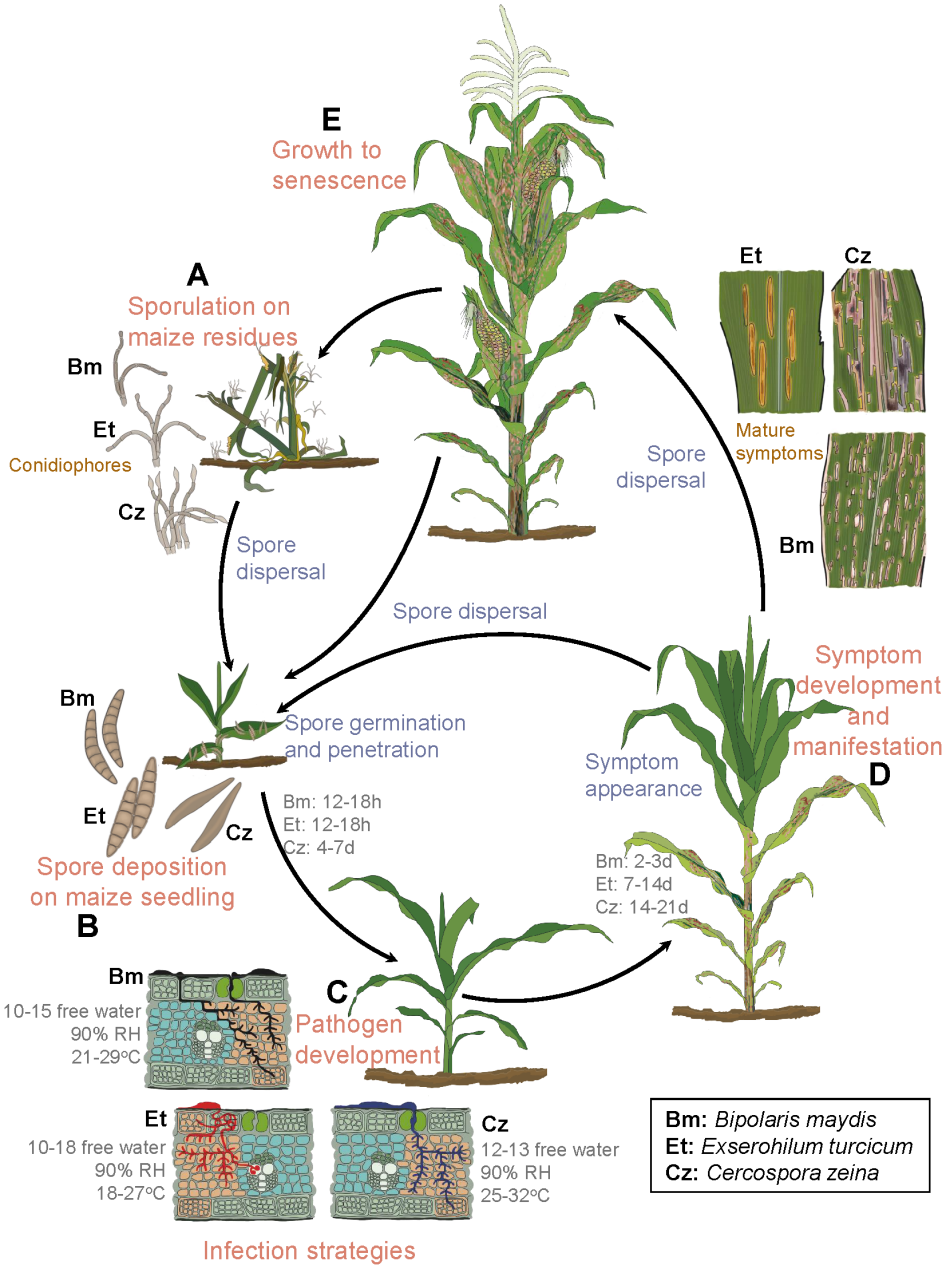
Asexual life cycles of *Exserohilum turcicum*, *Cercospora zeina*, and *Bipolaris maydis*. **(A)** Primary inoculum overwinters on maize debris as conidiophores until the next growing season, when they are dispersed in the form of conidia. **(B)** Under favorable conditions, conidia are dispersed and land on young maize plants. **(C)** Conidia germinate, penetrate plant cells, and later develop into small chlorotic spots. **(D, E)** Mature lesions develop from the lower leaves to younger leaves. These later give rise to conidia (secondary inoculum), which disperse to the younger plants, and the cycle repeats. Et, *E. turcicum* (Leonard, 1977b; Levy and Cohen, 1983; Bentolila et al., 1991; White, 1999; Kotze et al., 2019); Cz, *C. zeina* (Beckman and Payne, 1982; Latterell and Rossi, 1983; Ward et al., 1999; Wisser et al., 2011) and Bm, *B. maydis* (Jeffers, 2004; Wisser et al., 2011; Singh and Srivastava, 2012). Citations refer to sources for details of each pathogen’s disease cycle. **(C)** leaf cross sections were adapted from Supplementary Figure S1 of Wisser et al. (2011). The unit for free water is hours. RH = relative humidity.

**Figure 3:**
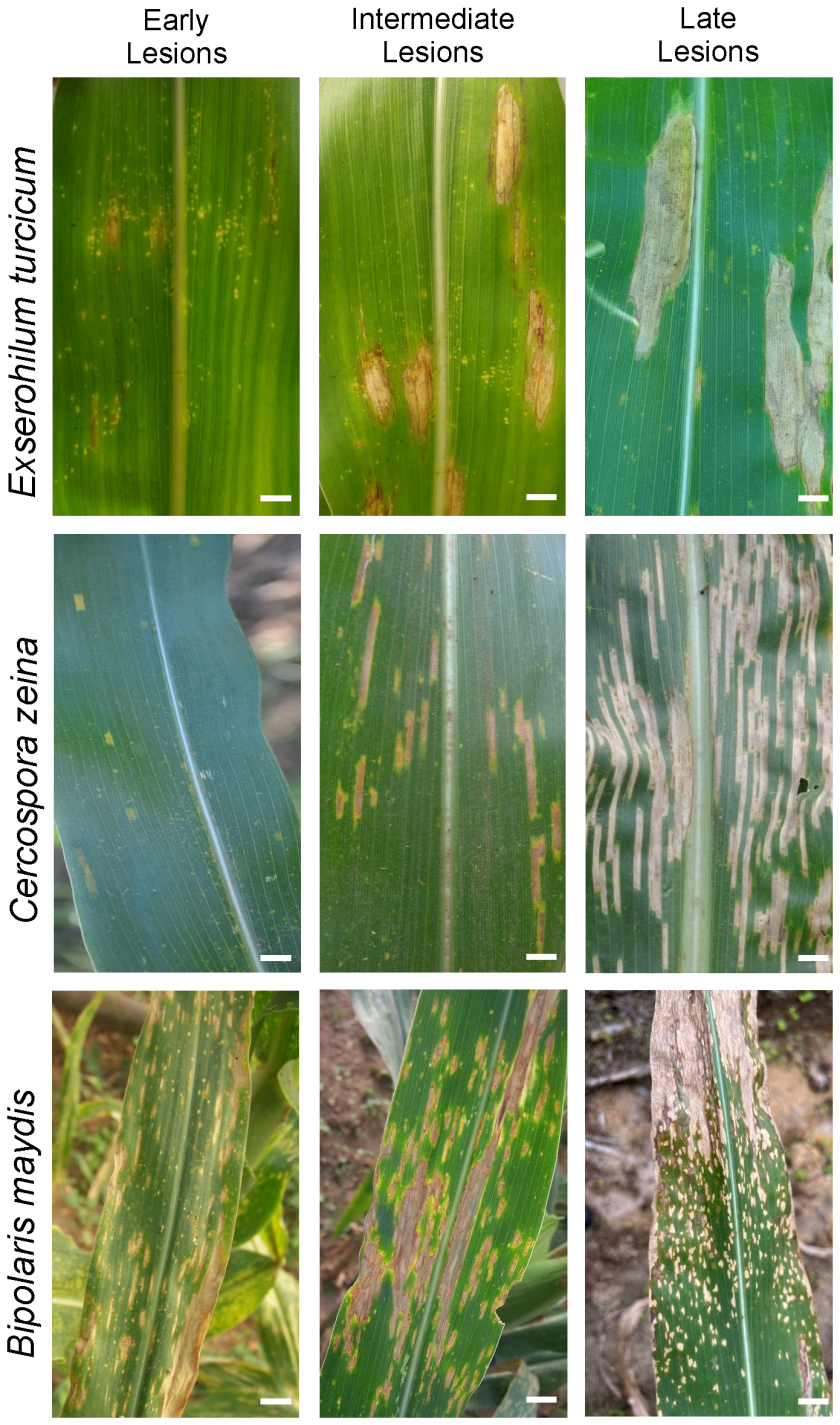
Symptomatic differences in NLB, GLS, and SCLB in maize. Each pathogen causes distinct disease symptoms during the intermediate and late stages of the infection cycle. Symptoms are prone to misidentification at early stages. All three diseases exhibit chlorotic spots in their early stages, making them difficult to diagnose. In the intermediate to late stages, each disease assumes its distinct lesion shape (i.e., cigar-shaped lesions for NLB, fine rectangular lesions for GLS, and rectangular lesions with irregular margins for SCLB symptoms, especially at the late stage). SCLB and GLS are not as clearly distinct as NLB. Scale bars = 2 cm.

Upon infection of maize leaves, *E. turcicum* causes greyish lesions that start as chlorotic flecks and later mature into elliptical or cigar-shaped lesions from 2.5 to 30 cm in length (White, 1999) (**Figures 2**, **3**). Disease establishment occurs within 6–18 h post-infection, and mature lesions develop within two weeks of host-pathogen interaction under favorable environmental conditions (Levy and Cohen, 1983; Bentolila et al., 1991; White, 1999; Kotze et al., 2019) (**Table 1**; **Figure 2**). The pathogen invades the host through the epidermis and blocks the vascular tissues (Kotze et al., 2019). This causes plant lodging and a reduction in photosynthetic leaf area, leading to 30%–91% yield losses in cases of severe infections during silking and grain filling (Tilahun et al., 2012; Nwanosike et al., 2015; Jindal et al., 2019; Berger et al., 2020). The NLB disease is a splash- and wind-borne polycyclic disease that spreads via conidia from infected debris left in the fields (**Figure 2**), and from secondary infections over long distances across fields (Schwartz and David, 2005).

### 3.2 Gray leaf spot

*Cercospora zeina* invades the host leaf tissues intracellularly resulting in irregular chlorotic lesions that after 14 days post infection, mature into grey to tan linear rectangular lesions that run parallel with leaf veins (Latterell and Rossi, 1983; Ward et al., 1999) (**Figures 2**, **3**). Extensive disease development results in the coalescence of the lesions, blighting, necrosis of the leaf tissue, reduced photosynthetic area and plant lodging (Paul and Munkvold, 2005; Lennon et al., 2016). The calculations made based on spore size (40 - 165 µm × 4 - 9 µm), wind speed (varies per location) and the height of vertical mixing of the atmosphere above the crop, estimate flight distances of spores to range between 0.1 - 40 km as wind speed increases from 1 to 10 m/s (Ward et al., 1999). The spores have also been reported to spread to a distance of 80 - 160 km annually, making it a fast-spreading disease (Manandhar et al., 2011). Yield losses due to GLS have been estimated to be 20–80% (Latterell and Rossi, 1983; Ward et al., 1999; Manandhar et al., 2011).

### 3.3 Southern corn leaf blight

Irrespective of race, *B. maydis* infections in maize generally take between 12 to 18 h for fungal penetration, and 2 to 3 days to form mature lesions (Singh and Srivastava, 2012) (**Figure 2**; **Table 2**). The race O causes small diamond-shaped lesions that elongate into rectangular lesions limited within veins to a length of 20-30 mm that later coalesce, resulting in the entire leaf blighting (Jeffers, 2004; Singh and Srivastava, 2012). The race T causes oval-shaped yellow to brown lesions that are larger than race O (Jeffers, 2004; Singh and Srivastava, 2012), and produces a T-cms-specific polyketide toxin (T toxin) that is specific to T-cms maize genotypes (Condon et al., 2018). This race, whose origin is still a mystery, was implicated in a serious epidemic in the USA in 1970 (Bruns, 2017). The SCLB disease thrives in hot and humid agroecosystems (Warren, 1975) (**Table 1**). Yield losses of 10–40% due to SCLB infections have been reported, depending on the physiological race, environment and the maize hybrid grown (Fisher et al., 2012; Bruns, 2017).

All three fungal pathogens infect the same plant parts (leaves). They have similar growth requirements of moderate temperatures between 20°C and 30°C with relative humidity above 90% favoring disease establishment. Yield losses from individual pathogens can be 10-80%. Therefore, there is a need to determine the impact of combined infections by measuring the percentage of co-occurrence of these three diseases on a plant, field, and larger spatial scale to model their combined potential yield losses. This will facilitate the development of management strategies that target both single and co-infections.

## 4 Diagnosis of NLB, GLS, SCLB and identification of their causal pathogens

Crop disease diagnosis and the identification of the causal organism up to species level are becoming more critical. This is because more disease epidemics are emerging globally as a result of increased anthropogenic activities, such as global trade and expansion of pathogen ranges due to climate change (Hulme, 2009; Elad and Pertot, 2014; Prakash et al., 2014; Chaloner et al., 2021). Failure to accurately diagnose diseases and correctly detect the causal pathogens leads to inadequate or delayed implementation of control measures, thus causing a reduction in crop yield and quality (Miller et al., 2009). Similar to other plant diseases, NLB, GLS, and SCLB and their corresponding causal pathogens, have been diagnosed based on symptoms, morphological characteristics, and molecular phylogenetics.

### 4.1 Field diagnosis of NLB, GLS, and SCLB

Traditionally, plant disease diagnosis is performed through conventional visual field inspection of infected plant tissues (symptoms) using experienced technical human resources (Bock et al., 2010). Two standard scales (1–5 and 1–9, where 1 = resistant and 5 or 9 = susceptible) are being used to rate the severity of NLB (Abebe et al., 2008; Asea et al., 2009; Vivek et al., 2010; Kumar et al., 2011), GLS (Bubeck et al., 1993; Munkvold et al., 2001; Danson et al., 2008; Chung et al., 2011; Berger et al., 2014; Benson et al., 2015), and SCLB (Zwonitzer et al., 2009; Chung et al., 2010, Chung et al., 2011; Singh and Srivastava, 2012) (**Table 2**). Some plant pathologists have preferred using the 1 to 9 scale in the field and later converted it to a scale of 1 to 5 using the following formula: 0.5 * (disease score (1 to 9 scale) + 1) = disease score (1 to 5 scale) (Vivek et al., 2010). These scales are used to assess disease severity over time which can be expressed as area under disease progress curve (AUDPC) or disease index (Ma et al., 2022). While this traditional method has been refined over time, it is plagued by the inherent subjectiveness of disease estimates and is time-consuming (Nutter et al., 1993; Bock et al., 2008; Poland and Nelson, 2011). Sometimes, morphological traits are misleading because of the similarities between disease symptoms. For instance, race O lesions of SCLB are sometimes mistaken for GLS, whereas the initial symptoms of NLB, GLS, and SCLB (chlorotic spots) can potentially lead to misidentification (**Figure 3**).

Digital imaging techniques based on standard RGB images or hyperspectral images captured manually with cameras, mobile phones, or captured automatically with drones or by satellites hold great promise for crop disease diagnostics (Mohanty et al., 2016; DeChant et al., 2017). These methods involve training computer models with datasets of disease images which have been pre-classified by plant pathologists. The models are then tested on new sets of disease images to evaluate the accuracy of diagnosis. Examples of machine learning methods that have been used for plant disease diagnosis are spectral angle mapper (SAM), partial least squares regression (PLSR), support vector machines (SVMs), and convolutional neural networks (CNNs) (Xie et al., 2012; Stewart and McDonald, 2014; Mutka and Bart, 2015; Pauli et al., 2016; MuLaosmanovic et al., 2020).

This is a very active area of research that is also being applied to maize foliar diseases such as NLB and GLS with accuracies of greater than 90%, but it is still in its infancy since most models are being trained on images with only one disease symptom type (Zhang, 2013; Xu et al., 2015; Mohanty et al., 2016; Qi et al., 2016; Singh et al., 2016; DeChant et al., 2017; Zhang et al., 2018; Craze et al., 2022; Pan et al., 2022). Attempts are underway to diagnose individual foliar diseases with more field-realistic images with multiple disease symptom types; for example a neural network model was developed to identify GLS symptoms on maize leaves which had mixed symptoms of NLB, common rust, and white spot disease (Craze et al., 2022).

These high-throughput diagnostic methods are a foundation for understanding pathogen ecology, epidemiology, and biology. Their integration into management programs for several plant diseases has the potential to foster a more targeted approach for the prevention of epidemics.

### 4.2 Morphological and physiological diagnosis and detection

**Figure 4:**
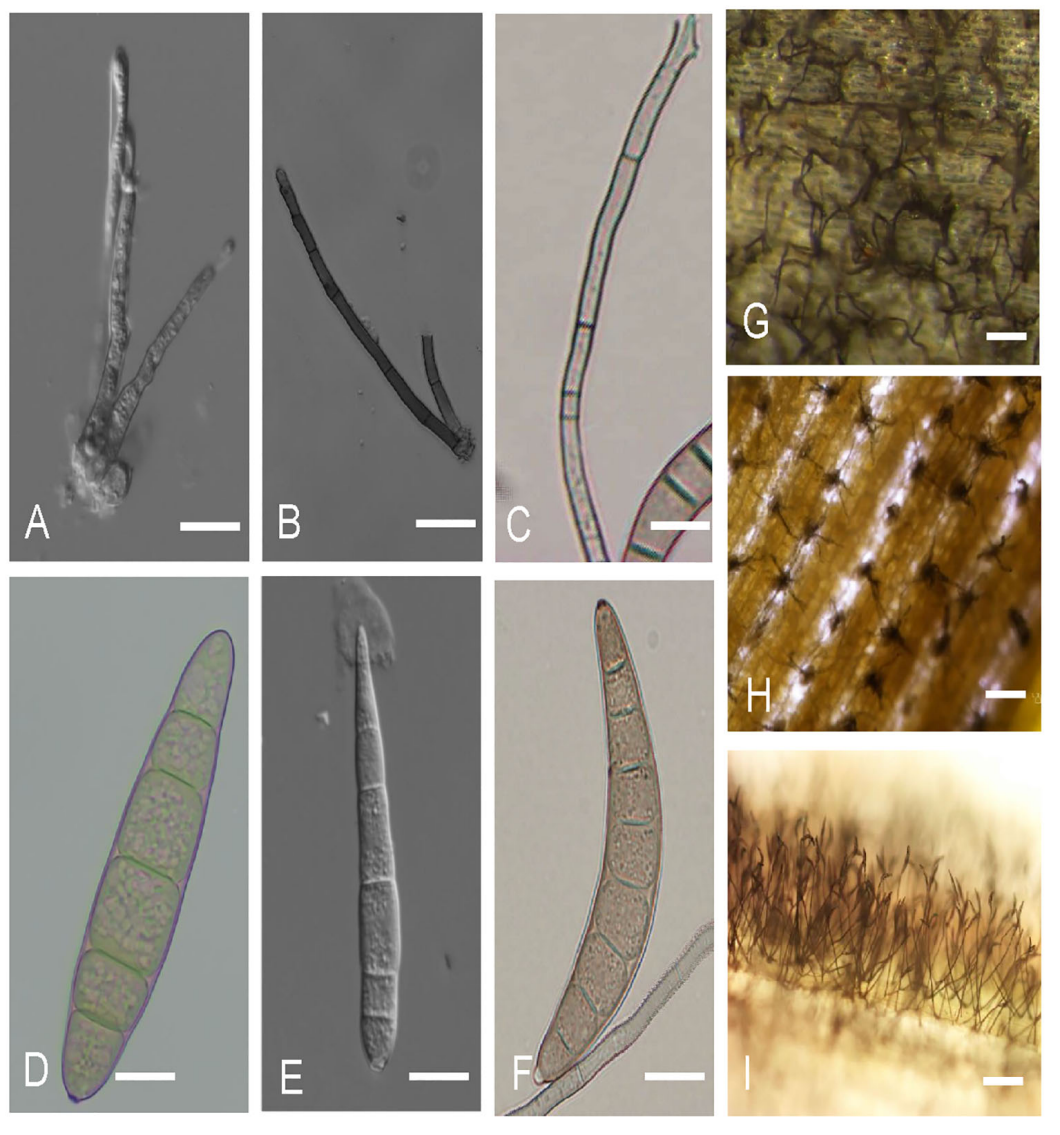
Asexual structures of *Exserohilum turcicum*, *Cercospora zeina*, and *Bipolaris maydis* in maize. **(A–C)** illustrates the conidiophores for **(A)** *E. turcicum*; **(B)** *C. zeina*; **(C)** *B. maydis*. **(D–F)** illustrate conidia of **(D)** *E. turcicum*; **(E)** *C. zeina*; **(F)** *B. maydis*. **(G–I)** Conidiophores of **(G)** *E. turcicum*, **(H)** *C. zeina* and **(I)** *B. maydis* on the surfaces of maize leaves Scale bars: **(A–F)** = 10 µm, **(G–I)** = 100 µm. **(C, F, I)** photos provided by Ms. Anu Elizabeth Ajayi, International Institute of Tropical Agriculture (IITA), Nigeria.

For years, pathogen identification has relied on conventional techniques such as culturing, re-inoculation, microscopy, and biochemical assays (Sharma and Sharma, 2016). Morphological methods, which depend on visible signs of post-fungal infections, such as symptoms and fungal propagules, can be used to distinguish between *E. turcicum* and *B. maydis*, based on a hilum. *Bipolaris maydis* has a subtle hilum (Alcorn, 1988), while *E. turcicum* has a truncated, prominent hilum with a bubble (Leonard, 1974) (**Figure 4**). Cercosporoid fungi, however, are mainly distinguished based on conidia, hila, and pigmentation of their asexual structures (Crous and Braun, 2003; Crous et al., 2006; Nsibo et al., 2021). *Cercospora zeina* conidia are characterized by their septate, hyaline, thin walls, smooth apex, and thick darkened and refractive hila (**Figure 4**). These characteristics are similar to those of *C. zeae-maydis*. However, they differ in conidia shape, conidiophore length, and growth rate (Crous et al., 2006). Furthermore, *C. zeae-maydis* produces a photoactive phytotoxin, cercosporin, *in vitro*, whereas *C. zeina* does not (Goodwin et al., 2001; Crous et al., 2006; Swart et al., 2017).

Disease diagnosis of NLB, GLS, and SCLB based on morphological differences of the fungal morphology is possible. However, this often requires isolation and culturing of the fungal pathogen, which is time-consuming. This makes these approaches inadequate for accurate and timely species-level identification (McCartney et al., 2003; Pryce et al., 2003).

### 4.3 Molecular identification

More advanced methods of identification, such as PCR-based amplification of nucleic acids and sequencing, are increasingly being employed for *E. turcicum*, *C. zeina*, and *B. maydis*. These methods can be more sensitive, are highly specific, faster, and require limited prior knowledge of the pathogen or expertise in plant pathology (McCartney et al., 2003; Ward et al., 2004). PCR amplification followed by sequencing of a fragment of the nuclear ribosomal DNAs (rDNAs), particularly the internal transcribed spacer (ITS), nested between conserved sequences of the 18S, 5.8S, and 28S rRNA gene regions, has been extensively employed for fungal species identification. The ITS marker is used as a universal barcode and is applicable for *E. turcicum* (Goh et al., 1998; Weikert-Oliveira et al., 2002; Ramathani et al., 2011; Haasbroek et al., 2014; Hernández-Restrepo et al., 2018), *C. zeina* (Dunkle and Levy, 2000; Crous et al., 2006; Meisel et al., 2009; Korsman et al., 2012; Liu and Xu, 2013; Bakhshi et al., 2015; Neves et al., 2015) and *B. maydis* (Goh et al., 1998; Emami and Hack, 2002; Manamgoda et al., 2012; Gogoi et al., 2014) (**Table 3**). The ITS region has multiple (identical) copies in the genome and it’s PCR products are small (less than 1 Kb) which allow for easy PCR amplification, even in dilute or partially degraded DNA (White et al., 1973; Gardes et al., 1991; Lee and Taylor, 1992; Schoch et al., 2012).

**Table 3:** The universal molecular bar codes used in the identification of the causal pathogens of NLB, GLS and SCLB.

Locus	Definition	Primer Name	Oligonucleotide (5’→3’)	Specificity	GenBank numbers			Reference
					*E. turcicum*	*C. zeina*	*B. maydis*	
**ITS**	Internal transcribed spacer region	ITS1	TCCGTAGGTGAACCTGCGG	Universal	NR_163537	DQ185081	NR_138224	Berbee et al., 1999; White, 1999; Meisel et al., 2009; Haasbroek et al., 2014; Tang et al., 2015
		ITS4	TCCTCCGCTTATTGATATGC	Universal				
**TEF1α**	Translation elongation factor 1 alpha	EF1-728F	CATCGAGAAGTTCGAGAAGG	Universal	LT896674	DQ185093	KM093793	Carbone and Kohn, 1999; Crous et al., 2006; Meisel et al., 2009; Neves et al., 2015
		EF1-986R	TACTTGAAGGAACCCTTACC	Universal				
**CAL**	Calmodulin	CAL-228F	GAGTTCAAGGAGGCCTTCTCCC	Universal	LT852468	DQ185117	HQ699077	Carbone and Kohn, 1999; Crous et al., 2006; Neves et al., 2015
		CAL-737R	CATCTTTCTGGCCATCATGG	Universal				
**ACT**	Actin	ACT-512F	ATGTGCAAGGCCGGTTTCGC	Universal	LT837686	DQ185105	AY748989	Carbone and Kohn, 1999; Crous et al., 2006
		ACT-783R	TACGAGTCCTTCTGGCCCAT	Universal				
**TUB**	β-tubulin	Bt1a	TTCCCCCGTCTCCACTTCTTCATG	Universal	LT899336	–	KX835024	Glass and Donaldson, 1995; Hernández-Restrepo et al., 2018
		Bt1b	GACGAGATCGTTCATGTTGAACTC	Universal				

These gene regions require PCR amplification, sequencing and phylogenetic analysis for species identification.

Other available DNA targets for pathogen identification include regions of the translation elongation factor 1-α, calmodulin, β-tubulin, glyceraldehyde-3-phosphate dehydrogenase and mating type genes (Carbone and Kohn, 1999; James et al., 2006; Walker et al., 2012). Most of these gene regions have been employed for the identification of *E. turcicum* (Ramathani et al., 2011; Haasbroek et al., 2014; Hernández-Restrepo et al., 2018), *C. zeina* (Meisel et al., 2009; Bakhshi et al., 2015; Muller et al., 2016; Nsibo et al., 2019, Nsibo et al., 2021), and *B. maydis* (Leonard, 1974; Turgeon et al., 1995; Manamgoda et al., 2012; Tan et al., 2016) in Africa and around the world (**Table 3**).

Various species-specific PCR diagnostic tools that do not require sequencing have been developed (**Table 4**). For *E. turcicum*, mating-type genes are currently the only available species-specific diagnostic method. The amplification of PCR products of 608 bp and 393 bp using either a *MAT1-1*F or *MAT1-2*F primer together with MAT_CommonR primer indicates the presence of *MAT1-1* or *MAT1-2*, respectively (Henegariu et al., 1997; Haasbroek et al., 2014) (**Table 4**).

**Table 4:** The species-specific molecular bar codes used in the identification of the causal pathogens of NLB, GLS and SCLB.

Locus	Definition	Primer name	Oligonucleotide (5’→3’)	Species	Amplicon size (bp)	Date published
CPR*	Cytochrome P450 reductase	CPR1_1F	TCCACTCTCGCTCAATTCG	*C. zeina*	164	2012
		CPR1_1R	GCCTTCATCGCCATATGTTC			
		CPR1_1F	TCCACTCTCGCTCAATTCG	*C. zeae-maydis*	164	
		CPR1_1R	GCCTTCATCGCCATATGTTC			
**CTB7**	Cercosporin toxin biosynthesis 7	CTB7-F	AAGAGTGCTTGTGAATGG	*C. zeina*	618	2017
		CTB7-R	GATGCGGGTGAAGTAGAAA			
		CTB7-F	AAGAGTGCTTGTGAATGG	*C. zeae-maydis*	925	
		CTB7-R	GATGCGGGTGAAGTAGAAA			
**HIST**	Histone H3	CylH3F	AGG TCC ACT GGT GGC AAG	*Cercospora* sp.	389	2004 and 2006
		CylH3R	AGC TGG ATG TCC TTG GAC TG			
		CzeinaHIST	TCGAGTGGCCCTCACCGT	*C. zeina*	284	
		CzeaeHIST	TCGACTCGTCTTTCACTTG	*C. zeae-maydis*	284	
		CmaizeHIST	TCGAGTCACTTCGACTTCC	*Cercospora* sp.	284	
**MAT**	Mating types	Cz*MAT1-1*F	TCACCCTTTCACCGTACCCA	*C. zeina*	631	2016
		Cz*MAT1-1*R	CACCTGCCATCCCATCATCTC			
		Cz*MAT1-2*F	CGATGTCACGGAGGACCTGA		409	
		Cz*MAT1-2*R	GTGGAGGTCGAGACGGTAGA			
		*MAT1-1*F	CTCGTCCTTGGAGAAGAATATC	*E. turcicum*	608	2014
		*MAT1-2*F	GCTCCTGGACCAAATAATACA		393	
		MAT_CommonR	AATGCGGACACGGAATAC			
		MAT113	AGGTAGTTTGAGGTGAGGGCAGATGATG	*B. maydis*	702	1997
		MATcon5	TCTTTGTTTTCCTGTGACTGCCTGTTG			
		MAT123	CTGGGCTGATTGGGGGCTTGATAC		547	
		MATcon5	TCTTTGTTTTCCTGTGACTGCCTGTTG			

*Primers can distinguish between *C. zeina* and *C. zeae-maydis* based on differences in their quantitative PCR (qPCR) melting peaks (Korsman et al., 2012).

A species-specific PCR diagnostic that can differentiate three maize *Cercospora* species (*C. zeina*, *C. zeae-maydis*, and *Cercospora* sp.) was based on the histone *H3* gene region (Crous et al. (2006). A multiplex PCR was used where universal primers CylH3F and CylH3R amplify a 389-bp fragment common to all three species. This universal primer pair is multiplexed with species-specific primers CzeaeHIST, CzeinaHIST, or CmaizeHIST in three separate PCR reactions for each unknown sample. Each reaction produces the common 389-bp fragment, and one of the three reactions will give a species-diagnostic 284-bp fragment (Crous et al., 2006) (**Table 4**). Limitations of this approach is the need to do three PCR reactions per sample, and the requirement for highly optimized PCR conditions to ensure only the correct species-specific primer binds to the target.

A cytochrome P450 reductase (*cpr1*) has been used to distinguish *C. zeina* and *C. zeae-maydis* from other maize pathogens. The CPR1_F and CPR1-R primers amplify a 164bp product from *C. zeina* and *C. zeae-maydis* but not from other maize pathogens (Korsman et al., 2012). Furthermore, the assay can also be used to differentiate *C. zeina* and *C. zeae-maydis* based on melting temperature differences between the products that can be measured after a real-time PCR reaction (Korsman et al., 2012).

Rapid identification of *C. zeina* or *C. zeae-maydis* is routinely carried out using primers in the cercosporin biosynthesis *CTB*7 gene region, since different sizes are produced for *C. zeina* compared to *C. zeae-maydis* (Swart et al., 2017; Nsibo et al., 2019, Nsibo et al., 2021). *Cercospora zeina* mating type markers amplify fragments that differentiate *C. zeina* MAT1-1 from MAT1-2 strains, with no amplification from species like *C. zeae-maydis* (Muller et al., 2016; Nsibo et al., 2019, Nsibo et al., 2021) (**Table 4**). For *B. maydis*, a multiplex mating-type PCR assay was optimized using primers MAT113, MAT123, and MATcon5 to amplify 702-bp (*MAT1-1*) and 547-bp (*MAT1-2*) fragments unique to *B. maydis* (Gafur et al., 1997) (**Table 4**).

Recently, high throughput diagnostics are being employed for the early detection of crop diseases. For example, nano-material-enabled sensors including carbon-based, metal- and metal oxide-based nanomaterials, are currently being used in the early detection of plant diseases based on the changes in the physiology of plants (Li et al., 2021). In addition, the RNA programmable nuclease of CRISPR/Cas is a nucleic acid detection tool that is currently being employed for crop disease diagnosis (Zhang et al., 2020; Wheatley et al., 2021; Liang et al., 2024). These methods are yet to be optimized for the detection of *E. turcicum*, *B. maydis* and *C. zeina*.

## 5 Genomic information for *Exserohilum turcicum*, *Cercospora zeina* and *Bipolaris maydis*

The development of molecular diagnostic tools and population genomics studies (see later) for these fungi will be increasingly supported in the future by genomics data, especially genome sequences. The first genome sequences that were available were developed using short-read Illumina sequencing, namely for USA strains of *E. turcicum* and *B. maydis* (Condon et al., 2013) and an African strain of *C. zeina* (CMW25467) from Zambia (Wingfield et al., 2017) (**Table 5**). Subsequently, these genome sequences were improved, for example, by including RNAseq data for better annotation, and using long read sequencing such as PacBio.

**Table 5:** Reference genome sequences.

Disease	Northern (corn) leaf blight	Northern (corn) leaf blight	Gray leaf spot	Southern corn leaf blight	Southern corn leaf blight
Old teleomorph name	*Setosphaeria turcica* Et28A v2.0 (race 23N)	*Setosphaeria turcica* NY001 v2.0 (race 1)	Unknown	*Cochliobolus heterostrophus* race T strain C4	*Cochliobolus heterostrophus* race O strain C5
Current species names	*Exserohilum turcicum*	*Exserohilum turcicum*	*Cercospora zeina* CMW 25467	*Bipolaris maydis* ATCC 48331	*Bipolaris maydis* ATCC 48332
Genome size (Mb)	43	38.4	41.7	37.79	36.5
Genome scaffold count	407	489	22	70	53
Scaffold N50 (length of scaffolds)	2.14 Mb	0.23 Mb	4 Mb	2.06	2.12 Mb
Scaffold L50 (# contigs in N50)	8	45	5	7	6
Repeat coverage (%)	13%	nd	AT rich component ~33%	13%	12%
Predicted genes	11702	12547	11570	11324	11808
Sequencing technology	Sanger, 454, Illumina	Illumina	PacBio	PacBio	PacBio
JGI version for this data	v2.0	v2.0	not available at JGI	v6.0	v4.0
JGI weblink	https://mycocosm.jgi.doe.gov/Settu3/Settu3.info.html	https://mycocosm.jgi.doe.gov/Settur3/Settur3.info.html	not available at JGI	https://mycocosm.jgi.doe.gov/CocheC5_4m/CocheC5_4m.info.html	https://mycocosm.jgi.doe.gov/CocheC5_4m/CocheC5_4m.info.html
GenBank Accession #	GCA_000359705.1 (v1.0)	not available at Genbank	MVDW02	GCF_000354255.1 (v1.0)	GCA_000338975.1 (v1.0)
Provenance of strain	USA	New York State, USA	Mkushi, Zambia	USA	USA
Reference	Condon et al., 2013 (v1.0)	Condon et al., 2013 (v1.0)	Wingfield et al., 2022	Haridas et al., 2023	Haridas et al., 2023

Details of the reference genome sequences for *E. turcicum*, *C. zeina*, *B. maydis* are presented in **Table 5**. It should be noted that some genome sequences are available from GenBank (https://www.ncbi.nlm.nih.gov/), whereas others are on the Mycocosm site at the Joint Genome Institute (JGI) (https://genome.jgi.doe.gov/portal/). Currently, the reference sequence for the NLB pathogen *S. turcica* (*E. turcicum*) Et28A race 23N and another USA strain NY001 race 1 are based on Illumina data (**Table 5**) (Condon et al., 2013). These assemblies resulted in genome sizes of 43 Mb and 38.4 Mb, respectively. However, due to short read sequencing it is likely the repetitive parts of these genomes are not fully assembled. The only genomics data set for an African isolate of *E. turcicum* is an *in planta* RNAseq time course for strain 2 (race 23N) and strain 103 (race 1) from South Africa (Human et al., 2020).

The reference genome for *C. zeina* is strain CMW25467 from Zambia in Africa (Wingfield et al., 2022). This high-quality genome sequence was determined using PacBio, resulting in 22 scaffolds (**Table 5**). In addition, Illumina genome sequences for 30 isolates of *C. zeina* from five countries in Africa were used for a population genomics study (Welgemoed et al., 2023). Recently, PacBio genomes for *C. heterostrophus* (*B. maydis*) race T strain C4 and race O strain C5 from the USA were reported (Haridas et al., 2023). These assemblies were 37.8 and 36.5 Mb in size in 70 and 53 scaffolds, respectively. Interestingly, the T-toxin biosynthetic cluster in race T was situated as dispersed genes within large stretches of repetitive DNA (Haridas et al., 2023). Overall, the number of predicted protein coding genes in these maize foliar pathogens were in a similar range of 11702 – 12547 (**Table 5**).

Genomic sequence information for these maize fungal pathogens opens several new avenues for disease control, as well as a deeper understanding of maize-pathogen interactions. Protein-coding gene catalogues of *E. turcicum*, *C. zeina*, and *B. maydis* can be searched for effector genes, known to be important for pathogenicity. Machine learning tools such as Effector P are used for this (Sperschneider and Dodds, 2022), as was done for *Bipolaris* spp. and *E. turcicum* (Condon et al., 2013; Human et al., 2020). Fungal effector genes interact with host proteins either directly or indirectly, and effector discovery is the first step in identifying host targets, eventually leading to effector-based breeding (Vleeshouwers and Oliver, 2014).

Gene catalogues for these maize pathogens can also prove useful for developing novel approaches for disease control, such as RNAi-based fungicides. In a recent study in *C. zeina*, a phylogenomic approach was used to first determine that this pathogen had the machinery for RNAi (Marais et al., 2024). This entailed comparing the protein-coding gene catalogue of 99 *Dothideomycetes* fungal genomes, and then drawing phylogenetic trees for orthogroups of Dicer-like, RNA-dependent RNA Polymerase and Argonaute. RNAi targets were identified in the *C. zeina* gene catalogue, allowing design of gene-specific dsRNAs. In a proof of concept, the dsRNA treatment disrupted the metabolic activity of the fungus *in vitro*, and reduced GLS disease when applied to inoculated maize leaves (Marais et al., 2024).

## 6 Management of NLB, GLS, and SCLB

Effective disease management strategies should aim to interfere with the most vulnerable stages of the pathogen life cycle to reduce the rate of disease development (Ward and Nowell, 1998; Shah and Dillard, 2010; Reddy et al., 2013). Cultural practices, chemical usage, and host genetic resistance are extensively employed in managing NLB, GLS, and SCLB.

### 6.1 Cultural practices for the control of NLB, GLS, and SCLB

Similar management strategies, including the use of tillage practices, rotation with non-host crops, and manipulation of environmental factors, are being used against NLB, GLS, and SCLB to reduce the amount of initial inoculum of the causal pathogens in the field (Ward and Nowell, 1998; Hooda et al., 2017). Deep tillage ensures the burial and destruction of pathogen inoculum in the soil (Payne and Waldron, 1983; Huff et al., 1988). Rotations for at least two years with non-host crops reduce fungal inoculum, especially in seasons with low disease incidence (Sharma and Payak, 1990; Ward and Nowell, 1998). In addition, manipulation of favorable environmental conditions (temperature, relative humidity, and leaf wetness) for pathogen development, especially early in the growing season, is crucial for hindering early season disease development (Ward and Nowell, 1998). Although these cultural practices are useful in managing these diseases and may be effective in low-risk areas, they are less effective when the disease is well-established (Ward et al., 1997; Lipps et al., 1998; Ward and Nowell, 1998).

### 6.2 Chemical control of NLB, GLS and SCLB

Broad-spectrum fungicides, specifically demethylation inhibitor (DMI), quinone outside inhibitor (QoI), and succinate dehydrogenase inhibitor (SDHI), are effective against NLB, GLS, and SCLB, especially in susceptible hybrids (Reddy et al., 2013; Weems and Bradley, 2017; Dai et al., 2018; Neves and Bradley, 2019; Sun et al., 2023). Furthermore, Iturin A2, a *Bacillus subtilis* compound, was developed into a fungicide effective against *B. maydis* and other fungi (Gong et al., 2006; Kim et al., 2010; Ye et al., 2012). Iturin A2 could potentially treat other maize pathogens like *E. turcicum* and *C. zeina* and should be tested. Despite fungicide effectiveness, resistance has developed in other cereal pathogens like *Zymoseptoria tritici*, *Pyrenophora teres* f. *teres*, and *Magnaporthe oryzae* (Bohnert et al., 2018; Ellwood et al., 2019; Garnault et al., 2019). A few fungicide sensitivity studies have been conducted on *E. turcicum* and *B. maydis* to DMI, QoI, and SDHI fungicides. To date, all have revealed high sensitivities to fungicides, with no resistance buildup yet (Chapara et al., 2012; Weems and Bradley, 2017; Yuli et al., 2017; Hou et al., 2018). However, Africa lacks baseline sensitivity studies on *E. turcicum*, *C. zeina*, and *B. maydis*, despite increasing fungicide demands and use in large-scale field plantings. These studies are needed before fungicide resistance monitoring in maize foliar pathogens is initiated in Africa. Chemical control is also too expensive for many smallholder farmers. Therefore, affordable and long-lasting strategies such as host resistance through breeding need to be integrated and utilized.

### 6.3 Breeding for resistance against NLB, GLS and SCLB

Host plant resistance is the most economical, eco-friendly, and adjustable approach for maize disease management. Nelson et al. (2018) pointed out that effective resistance depends on the effect and strength of resistance genes in the host. Major genes provide complete or near-complete resistance, while quantitative resistance involves multiple minor genes with small additive effects (St. Clair, 2010).

### 6.4 Qualitative breeding for resistance

Resistance to *E. turcicum* in maize is both qualitative and quantitative and can be used either separately or in combination with qualitative resistance following a gene-to-gene model (Welz and Geiger, 2000; Ogliari et al., 2005) (**Table 2**). Qualitative resistance is mediated by *Helminthosporium turcicum* (*Ht*) resistance genes (Welz and Geiger, 2000). The four well-known *Ht* genes include *Ht1, Ht2, Ht3*, and *Htn1*, where the functions of *Ht1, Ht2* and *Ht3* have yet to be characterized (Van Staden et al., 2001; Yin et al., 2003; Ogliari et al., 2005). The *Htn1* gene is highly conserved in *E. turcicum* hosts, particularly maize, sorghum, rice, and foxtail millet (*Setaria italica*), and encodes a wall-associated receptor-like kinase that confers resistance against race 12 (Hurni et al. (2015). Other resistance genes include *HtP* against races 123x and 23rx (Ogliari et al., 2005) and the recessive genes *ht4* and *rt* that confer resistance to a wide range of *E. turcicum* races (Ogliari et al., 2005).

For GLS, a qualitative resistance locus is yet to be characterized. To date, GLS resistance is qualitatively inherited. Few major resistance quantitative trait loci including *Qgls*8 derived from teosinte (Zhang et al., 2017), and *gRgls*1 and *qRgls*2 from maize (Zhang et al., 2012) have been precisely defined.

Qualitative genes can confer resistance to *B. maydis* races. For instance, *rhm* gene mainly protects maize against race O and, to a lesser extent, race T strains (Zaitlin et al., 1993; Chang and Peterson, 1995). Chang and Peterson (1995) proposed a two-gene model in which two homozygous recessive genes, *rhm1* and *rhm2* which, in combination, increased host resistance to *B. maydis*. This was confirmed by the reduced lesion size as compared to the control experiment (Chang and Peterson, 1995) or the effect of an individual gene (Simmons et al., 2001). Qualitative resistance is effective against race O, while the best defense against race T is to avoid T-cms maize germplasm in breeding programs (Leonard, 1977a). Resistance to C and S races is so far unknown (Rong and Baxter, 2006).

### 6.5 Quantitative breeding for resistance

Quantitative disease resistance is known to reduce disease severity and incidence, rather than completely eliminate the disease (Young, 1996; Poland et al., 2009). In recent years, QTL mapping studies have characterized several traits of crops, including resistance to several plant pathogens (Bernardo, 2008; Xu and Crouch, 2008).

Quantitative trait loci for resistance against NLB span the entire maize genome and have been identified in several mapping populations (Welz and Geiger, 2000; Wisser et al., 2006; Chen et al., 2016; Wang et al., 2018; Wende et al., 2018; Rashid et al., 2020). Using techniques such as genome-wide nested association mapping, QTLs with several potential candidate genes have been characterized and confirmed to confer resistance against NLB (Poland et al., 2011; Rashid et al., 2020). Although many QTLs are known to confer resistance to a broad spectrum of *E. turcicum* races, some QTLs are known to confer race-specific resistance to NLB (Chung et al., 2010, Chung et al., 2011).

Hot spots of QTLs conferring resistance to GLS span discrete regions of chromosomes 1, 2, 3, 4, 5, and 7 (Lehmensiek et al., 2001; Berger et al., 2014). Most notably, a candidate gene encoding a maize caffeoyl-CoA O-methyltransferase that confers quantitative resistance to GLS and SCLB has been cloned, implicating lignin and the phenylpropanoid pathway in maize defense against foliar diseases (Yang et al., 2017).

Many of these QTLs are derived from bi-parental crosses between susceptible and resistant genotypes tested under different disease pressures, germplasm backgrounds, and environmental conditions (Clements et al., 2000; Lehmensiek et al., 2001; Balint-Kurti et al., 2008; Berger et al., 2014). A majority of the QTLs are environment-specific; however, many QTLs expressed in several environments have also been characterized (Berger et al., 2014). These can be introgressed into maize genotypes grown in different environments. Molecular breeding to develop GLS resistant maize for small-holder farmers in Africa has been reported (Kibe et al., 2020). Recently, advanced populations of maize developed by CIMMYT were used in a combination with linkage and association mapping with genome wide SNP markers to identify QTLs for GLS and NLB resistance in East Africa (Omondi et al., 2023).

Using recombinant inbred lines (RILs), Carson et al. (2004) identified 11 QTLs spanning chromosomes 1, 2, 3, 4, 7, and 10, which are associated with SCLB resistance. Additional SCLB resistance QTLs have been characterized from different maize genotypes at different maturity stages (Balint-Kurti and Carson, 2006; Zwonitzer et al., 2009; Negeri et al., 2011).

Thus, qualitative, and quantitative resistance breeding are crucial for managing NLB, GLS, and SCLB. Maize geneticists have made great progress in identifying the genomic loci associated with resistance to one or more of these three diseases. To identify these loci tools such as the nested association mapping (NAM) panel, and the availability of genome wide SNP markers (Benson et al., 2015) have been employed. Importantly, some loci appear to confer multiple disease resistance, and recent advances have validated some QTL in independent maize populations (Lopez-Zuniga et al., 2019). A limitation is that most of these studies have been carried out in the USA and Europe often with inbred lines adapted to these temperate climates, with disease scoring carried out against local populations of each pathogen (Technow et al., 2013). Molecular marker-assisted breeding tools can now be employed to introgress these resistance alleles into germplasm adapted to maize-producing regions in Africa to determine whether crop protection is conferred against pathogen populations on the continent.

## 7 Recent advances in population genetics of the causal pathogens of NLB, GLS, and SCLB

Pathogen survival is based on its ability to adapt to constant environmental changes through evolution (McDonald, 1997). Therefore, management strategies to counteract these fast-changing lifestyles must be guided by understanding the genetics of populations and their evolution in response to changing environments rather than a focus on individual “model” pathogen strains (McDonald, 1997).

### 7.1 Population genetics of *Exserohilum turcicum*

Microsatellite markers have replaced earlier techniques such as Random Amplified Polymorphic DNA (RAPD) and Amplified Fragment Length Polymorphism (AFLP) markers to study the global population structure of *E. turcicum*. Reports from Asia, Europe, the Americas, and Africa show that *E. turcicum* is genetically and genotypically diverse, with higher diversity in Asia and Africa (Borchardt et al., 1998b; Ferguson and Carson, 2004; Dong et al., 2008; Haasbroek et al., 2014; Tang et al., 2015; Nieuwoudt et al., 2018). European *E. turcicum* populations are characterized by low genetic diversity (Borchardt et al., 1998a; Turgay et al., 2021) and are partially differentiated due to the Alps (Borchardt et al., 1998a). African geographic boundaries, like mountains and the large lakes of the Rift Valley, may affect *E. turcicum* population structure, although this has not yet been investigated. Sexual recombination is a major evolutionary factor in *E. turcicum’s* global population structure, even in Europe, where sexual occurrences are rare, based on the frequency distribution of mating types and a lack of observed sexual structures in nature or in the laboratory (Fan et al., 2007; Turgay et al., 2021). Mating-type genes were found to be equally distributed and frequent in several countries where mating-type studies have been conducted, except in Europe, indicating sexual recombination (Haasbroek et al., 2014; Human et al., 2016; Weems and Bradley, 2018). Even though *S. turcica*, the sexual stage of *E. turcicum* is very rare in nature, with the only existing report being from Thailand (Bunkoed et al., 2014), it has been induced under laboratory conditions using Sach’s medium with barley culm (Moghaddam and Pataky, 1994; Fan et al., 2007).

Population genetic analysis allows for the study of physiological race distribution, potential race re-emergence, and the identification of alternative hosts. However, limited knowledge exists on the population genetic diversity and race diversity of *E. turcicum* in Africa, with the exception of populations in Kenya and South Africa. Therefore, understanding the population structure of *E. turcicum* in maize-growing African countries is needed.

### 7.2 Population genetics of *Cercospora zeina*

Prior to the classification of the GLS causal pathogens into two distinct species (Crous et al., 2006), all studies conducted on GLS referred to the disease as being caused by *C. zeae-maydis* (Latterell and Rossi, 1983; Lipps, 1998; Ward et al., 1999; Okori et al., 2003). As more studies based on taxonomy, molecular and phylogenetic tools emerged, it became evident that there were two sibling species, *C. zeina* (formerly known as *C. zeae-maydis* type II) and *C. zeae-maydis*. Initial molecular studies of *Cercospora* isolated from maize in Africa indicated the presence *C. zeae-maydis* type II (*C. zeina*) using AFLP and RFLP markers with genetic relatedness to Type II, and not Type 1 isolates in the Americas (Wang et al., 1998; Dunkle and Levy, 2000; Okori et al., 2003; Liu and Xu, 2013; Muller et al., 2016). Subsequent surveys in sub-Saharan Africa confirmed that *C. zeina* was the only causal pathogen in Africa since to date, no isolates of *C. zeae-maydis* have been found in a collection of more than 1000 isolates from East and Southern Africa (Nsibo et al., 2021).

Populations studies have shown that *C. zeina* is a highly diverse pathogen in Africa with a partially defined population structure within and among countries (Okori et al., 2003, Okori et al., 2015; Muller et al., 2016; Nsibo et al., 2019, Nsibo et al., 2021). Given that maize is non-native to Africa, the dominance of *C. zeina* on the continent is attributed to more than one introduction, followed by several sexual recombination events, intra-continent gene flow, migration, and local adaptations (Nsibo et al., 2021). This work was further refined by genome sequencing of 30 isolates of *C. zeina* from two countries in East Africa (Kenya, Uganda) and three countries in Southern Africa (Zambia, Zimbabwe and South Africa) (Welgemoed et al., 2023). This showed population differentiation but no major differences in diversity indices between regions, indicating two possible introductions over the approximately 500-year time period since maize entered the continent (Welgemoed et al., 2023). The study of *C. zeina* populations from other continents and the search for alternate hosts is underway to explore alternative hypotheses, including the hypothesis that *C. zeina* in Africa was derived from a host jump from an unidentified grass species onto maize (Dunkle and Levy, 2000; Crous et al., 2006).

Although sexual structures of *C. zeina* have not been observed under field and laboratory conditions, cryptic sexual recombination has been suggested based on the presence of mating-type genes with equal distribution and frequency, in addition to the low levels of linkage disequilibrium among some populations (Groenewald et al., 2006; Muller et al., 2016; Nsibo et al., 2021). Possible explanations for the failure of laboratory experiments to induce or discover the sexual stage of *C. zeina* may include the absence of environmental parameters that the pathogen encounters in nature to trigger sexual recombination. It could also be a failure to systematically monitor the development of an ascocarp in this presumably asexual pathogen (Dyer et al., 1992) or fertility decline in the pathogen (Dyer and Paoletti, 2005).

There is clear evidence that *C. zeina* is a well-established pathogen in Africa with the potential to threaten food production on the continent if not monitored to determine its diversity and migration patterns and deploy more effective management strategies.

### 7.3 Population genetics of *Bipolaris maydis*

There is limited information regarding the genetic diversity of *B. maydis*. RAPD markers have been used to understand the genetic structure of *B. maydis* populations, especially in India, where most reports have emerged. In India, *B. maydis* has been reported to be highly diverse, with little to no population differentiation (Karimi, 2003; Jahani et al., 2011; Gogoi et al., 2014), suggesting that gene flow plays a major evolutionary role in the population structure of the pathogen. Furthermore, the physiological race O is the most predominant race in India (Gogoi et al., 2014; Pal et al., 2015), with high genetic variability among isolates of the same race (Gafur et al., 2002; Pal et al., 2015). Sexual recombination is another major evolutionary factor driving observed genetic diversity (Gafur et al., 1997, Gafur et al., 2002). The availability of the *B. maydis* genome (Condon et al., 2013) offers a unique opportunity to develop more robust molecular markers, such as microsatellite and single-nucleotide polymorphism (SNP) markers, that can be exploited to enable comprehensive studies of the pathogen from all the countries where the disease exists.

*Bipolaris maydis* is a potential threat to maize production in Africa although it has only been reported in Kenya (Mwangi, 1998) and South Africa (Rong and Baxter, 2006). The risk of its spreading to other countries is heightened by the fact that *B. maydis* is both an air- and seed-borne pathogen (Aylor and Lukens, 1974; Manoj and Agarwal, 1998; Biemond et al., 2013). Due to increasing anthropogenic activities and global trade, unreported incidences of the pathogen in the rest of Africa are possible. Therefore, countries where SCLB has not yet been reported must be vigilant through the establishment of phytosanitary regulations and bodies that test and ensure the movement of healthy seeds across geographical boundaries. Methods such as roguing, seed dressing, and proper storage to minimize contamination have been suggested as alternative ways to ensure seed health (Biemond et al., 2013).

## 8 Breeding for multiple disease resistance against NLB, GLS and SCLB

Qualitative and quantitative disease resistance strategies, either individually or in combination (McDonald and Linde, 2002), are important for the management of NLB, GLS, and SCLB. These strategies are based on the development of advanced maize genetic populations and screening for disease resistance across multiple environments (see examples in next section). Multi-environment field testing aims to expose the maize populations with the “diversity” of pathogen genotypes (i.e. races). This, however, can now be done more systematically by supplementing the pathogen diversity by artificial inoculation if pathogen population genetics and race typing data are available.

The co-occurrence of maize foliar diseases such as NLB, GLS and SCLB in some maize production regions of Africa presents an additional challenge for maize breeders. As described above, many resistance QTL are available for each disease, however each locus may have a small effect and thus breeders need to introgress several QTL for durable resistance (Nelson et al., 2018). Researchers have, therefore, searched for multiple disease resistance loci in maize and other plants (Wiesner-Hanks and Nelson, 2016).

A multiple disease resistance QTL associated with resistance to NLB, GLS and SCLB was identified by association mapping with a panel of 253 genetically diverse maize genotypes that had been scored for each disease in the field (Wisser et al., 2011). Moderate resistance to all three diseases was found to be associated with alleles of a maize glutathione S-transferase (GST) gene (Dean et al., 2005; Wisser et al., 2011). This maize GST may play a general defense role against all three diseases through detoxification of fungal secondary metabolites.

In another study, quantitative resistance to both GLS and SCLB was associated with alleles of the *ZmCCoAOMT2 gene*, which encodes a caffeoyl-CoA *O*-methyltransferase (Yang et al., 2017). This enzyme is involved in the phenylpropanoid pathway and lignin production, thus potentially contributing to defense barriers against the invading fungal pathogens. In another study, the search for robust QTL for resistance to NLB and SCLB was carried out using the maize Nested Associated Mapping (NAM) panel, high density markers, and field testing in the USA and China. Some of the identified QTLs conferred resistance to both NLB and SCLB, and one of the candidate genes was *ZmCCoAOMT2*, providing validation of the previous finding (Li et al., 2018).

Backcross populations developed between four multiple disease resistant and two susceptible maize lines were used to identify several QTLs associated with resistance to NLB, GLS, and/or SCLB (Lopez-Zuniga et al., 2019). Several of these QTL conferred resistance to two of the diseases, and six to all three (NLB, GLS and SCLB) (Lopez-Zuniga et al., 2019). Further work validated these QTL by developing populations in more uniform genetic backgrounds, and two QTL were confirmed to be associated with resistance to all three diseases (Martins et al., 2019).

A take home message from these studies is that quantitative resistance to each of these three foliar diseases is generally conferred by many QTL in a single maize genotype, each with minor but additive effects (Li et al., 2018). In addition, QTL conferring resistance to more than one disease are rare and would be limited to common responses to the different pathogens (Martins et al., 2019).

Pyramiding qualitative resistance genes has been successful in other cereal pathosystems, such as wheat against the Ug99 races of stem rust (Zhang et al., 2019). However, combining QTLs for Ug99 resistance has been proposed as the most durable strategy for resistance (Singh et al., 2006). Pyramiding QTL would be the most durable strategy in maize against NLB, GLS, and SCLB when integrated with other management strategies.

Recent findings about both qualitative resistance genes like the Ht genes and multiple disease resistance QTLs described above have potential to benefit maize disease resistance breeders in Africa. However, several factors need to be fulfilled namely (i) access to germplasm and research capacity, (ii) efficacy of resistance loci in local environments, and (iv) ongoing research into pathogen population dynamics across the continent (Nsibo et al., 2021). Understandably, tightened phytosanitary regulations limits the ease with which maize germplasm can be shipped around the world. Fortunately, many African countries have historical access to maize germplasm through national breeding programmes, seed companies or NGOs such as the CGIAR institutes (Berger et al., 2020; Kibe et al., 2020). There are ongoing successes in releasing stress-tolerant maize varieties to farmers in Africa, which provides a good foundation to build on (Worku et al., 2020).

This highlights the importance of regional and international networks to address the threat of these three maize foliar diseases in Africa. Regional collaboration is important since fungal pathogens do not respect country borders. A successful example is the ongoing collaboration between universities in South Africa and Kenya which started with surveillance of the GLS pathogen *C. zeina* (Nsibo et al., 2021). Subsequently, CIMMYT came on board to expand the project to maize disease resistance breeding (Omondi et al., 2023). The collaboration had a strong component of capacity building with maize foliar disease workshops and postgraduate exchanges and training. The network is now supporting new maize disease reports in other countries of southern Africa. International linkages are key, for example collaboration with a German University has brought in whole genome-based population genomics expertise to the *C. zeina* project (Welgemoed et al., 2023), and funding from the British Society for Plant Pathology has facilitated expansion of the project to *E. turcicum*.

## 9 Conclusion

This review summarizes recent advances in NLB, GLS, and SCLB disease resistance breeding, as well as the ecology and population genetics of their causal pathogens in Africa. All three diseases exist on the continent and threaten its maize production and food security. These diseases are polycyclic in nature and can infect maize under overlapping environmental conditions within a single growing season. With the increasing adoption of conservation agriculture and monocropping, foliar diseases are likely to escalate to all maize-producing countries owing to the accumulation of inoculum and shared dispersal mechanisms. Several management strategies at the commercial level, particularly cultural practices, fungicide usage, and breeding for resistance, are being increasingly adopted and used in Africa. However, since most farming is on a small scale, fungicide usage is not widespread due to its cost implications and its aftereffects on soil and human health. As such, there is an increasing adoption of breeding for resistance at a small-scale level, used in combination with cultural practices. Notably, limited knowledge is available on the population biology and genetics of *E. turcicum*, *C. zeina*, and *B. maydis* in Africa; thus, the evolutionary potential of these pathogens to overcome resistance has not been fully established. Therefore, there is a need to conduct large-scale sampling of isolates across the continent to study their diversity and trace their migration patterns across the continent.

## Author contributions

DN: Data curation, Formal analysis, Investigation, Methodology, Software, Validation, Visualization, Writing – original draft, Writing – review & editing. IB: Conceptualization, Data curation, Formal analysis, Resources, Software, Supervision, Validation, Writing – review & editing. DB: Funding acquisition, Investigation, Methodology, Project administration, Resources, Supervision, Validation, Visualization, Writing – review & editing.

## References

[B1] Abadi R. Levy R. Levy Y. (1993). Mating types of *Exserohilum turcicum* in Israel. Phytoparasitica 21, 315–320. doi: 10.1007/BF02981049

[B2] Abadi R. Levy Y. Bar-Tsur A. (1989). Physiological races of *Exserohilum turcicum* in Israel. Phytoparasitica 17, 23–30. doi: 10.1007/BF02979602

[B3] Abebe D. Singburaudom N. (2006). Morphological, cultural and pathogenicity variation of *Exserohilum turcicum* (pass) Leonard and Suggs isolates in maize (*Zea mays* L.). Kasetsart J. Natural Sci. 40, 341–352.

[B4] Abebe D. Singburaudom N. Sangchote S. Sarobol E. (2008). Evaluation of maize varieties for resistance to northern leaf blight under field conditions in Ethiopia. Kasetsart J. Natural Sci. 42, 1–10.

[B5] Adipala E. Takan J. Ogenga-Latigo M. (1995). Effect of planting density of maize on the progress and spread of northern leaf blight from *Exserohilum turcicum* infested residue source. Eur. J. Plant Pathol. 101, 25–33. doi: 10.1007/BF01876091

[B6] Alcorn J. (1988). The taxonomy of "*Helminthosporium*" species. Annu. Rev. Phytopathol. 26, 37–56. doi: 10.1146/annurev.py.26.090188.000345

[B7] Aregbesola E. Ortega-Beltran A. Falade T. Jonathan G. Hearne S. Bandyopadhyay R. (2020). A detached leaf assay to rapidly screen for resistance of maize to *Bipolaris maydis*, the causal agent of southern corn leaf blight. Eur. J. Plant Pathol. 156, 133–145. doi: 10.1007/s10658-019-01870-4

[B8] Asea G. Vivek B. S. Bigirwa G. Lipps P. E. Pratt R. C. (2009). Validation of consensus quantitative trait loci associated with resistance to multiple foliar pathogens of maize. Phytopathology 99, 540–547. doi: 10.1094/PHYTO-99-5-0540 19351250

[B9] Aylor D. E. Lukens R. J. (1974). Liberation of *Helminthosporium maydis* spores by wind in the field. Phytopathology 64, 1136–1138. doi: 10.1094/Phyto-64-1136

[B10] Bakhshi M. Arzanlou M. Babai-Ahari A. Groenewald J. Z. Crous P. W. (2015). Is morphology in *Cercospora* a reliable reflection of generic affinity. Phytotaxa 213, 022–034. doi: 10.11646/phytotaxa.213.1

[B11] Balint-Kurti P. Carson M. (2006). Analysis of quantitative trait loci for resistance to southern leaf blight in juvenile maize. Phytopathology 96, 221–225. doi: 10.1094/PHYTO-96-0221 18944435

[B12] Balint-Kurti P. J. Wisser R. Zwonitzer J. C. (2008). Use of an advanced intercross line population for precise mapping of quantitative trait loci for gray leaf spot resistance in maize. Crop Sci. 48, 1696–1704. doi: 10.2135/cropsci2007.12.0679

[B13] Balint-Kurti P. Zwonitzer J. C. Wisser R. J. Carson M. Oropeza-Rosas M. A. Holland J. B.. (2007). Precise mapping of quantitative trait loci for resistance to southern leaf blight, caused by *Cochliobolus heterostrophus* race O, and flowering time using advanced intercross maize lines. Genetics 176, 645–657. doi: 10.1534/genetics.106.067892 17339203 PMC1893051

[B14] Bashir K. Kamaruzaman S. Khairulmazmi A. (2018). First report of northern corn leaf blight disease caused by *Exserohilum turcicum* on *Zea mays* in Malaysia. J. Mol. Genet. Med. 12, 1747–0862.1000387. doi: 10.4172/1747-0862.1000387

[B15] Bateman G. Gutteridge R. Gherbawy Y. Thomsett M. Nicholson P. (2007). Infection of stem bases and grains of winter wheat by *Fusarium culmorum* and *F. graminearum* and effects of tillage method and maize-stalk residues. Plant Pathol. 56, 604–615. doi: 10.1111/j.1365-3059.2007.01577.x

[B16] Bebber D. P. (2015). Range-expanding pests and pathogens in a warming world. Annu. Rev. Phytopathol. 53, 335–356. doi: 10.1146/annurev-phyto-080614-120207 26047565

[B17] Beckman P. M. Payne G. A. (1982). External growth, penetration, and development of *Cercospora zeae-maydis* in corn leaves. Phytopathology 72, 810–815. doi: 10.1094/Phyto-72-810

[B18] Benson J. M. Poland J. A. Benson B. M. Stromberg E. L. Nelson R. J. (2015). Resistance to gray leaf spot of maize: genetic architecture and mechanisms elucidated through nested association mapping and near-isogenic line analysis. PloS Genet. 11, e1005045. doi: 10.1371/journal.pgen.1005045 25764179 PMC4357430

[B19] Bentolila S. Guitton C. Bouvet N. Sailland A. Nykaza S. Freyssinet G. (1991). Identification of an RFLP marker tightly linked to the *Ht1* gene in maize. Theor. Appl. Genet. 82, 393–398. doi: 10.1007/BF00588588 24213251

[B20] Berbee M. Pirseyedi M. Hubbard S. (1999). *Cochliobolus* phylogenetics and the origin of known, highly virulent pathogens, inferred from ITS and glyceraldehyde-3-phosphate dehydrogenase gene sequences. Mycologia 91 (6), 964–977. doi: 10.1080/00275514.1999.12061106

[B21] Berger D. K. Carstens M. Korsman J. N. Middleton F. Kloppers F. J. Tongoona P.. (2014). Mapping QTL conferring resistance in maize to gray leaf spot disease caused by *Cercospora zeina* . BMC Genet. 15, 60. doi: 10.1186/1471-2156-15-60 24885661 PMC4059882

[B22] Berger D. K. Mokgobu T. Ridder K. Christie N. Aveling T. A. (2020). Benefits of maize resistance breeding and chemical control against northern leaf blight in smallholder farms in South Africa. South Afr. J. Sci. 116, 1–7. doi: 10.17159/sajs.2020/8286

[B23] Bernardo R. (2008). Molecular markers and selection for complex traits in plants: learning from the last 20 years. Crop Sci. 48, 1649–1664. doi: 10.2135/cropsci2008.03.0131

[B24] Biemond P. Oguntade O. Stomph T.-J. Kumar P. L. Termorshuizen A. J. Struik P. C. (2013). Health of farmer-saved maize seed in north-east Nigeria. Eur. J. Plant Pathol. 137, 563–572. doi: 10.1007/s10658-013-0269-5

[B25] Bock C. Parker P. Cook A. Gottwald T. (2008). Visual rating and the use of image analysis for assessing different symptoms of citrus canker on grapefruit leaves. Plant Dis. 92, 530–541. doi: 10.1094/PDIS-92-4-0530 30769647

[B26] Bock C. Poole G. Parker P. Gottwald T. (2010). Plant disease severity estimated visually, by digital photography and image analysis, and by hyperspectral imaging. Crit. Rev. Plant Sci. 29, 59–107. doi: 10.1080/07352681003617285

[B27] Bohnert S. Heck L. Gruber C. Neumann H. Distler U. Tenzer S.. (2018). Fungicide resistance toward fludioxonil conferred by overexpression of the phosphatase gene *ΔMoPTP2* in *Magnaporthe oryzae* . Mol. Microbiol. 111 (3), 662–677. doi: 10.1111/mmi.14179 30537256

[B28] Borchardt D. S. Welz H. G. Geiger H. H. (1998a). Genetic structure of *Setosphaeria turcica* populations in tropical and temperate climates. Phytopathology 88, 322–329. doi: 10.1094/PHYTO.1998.88.4.322 18944955

[B29] Borchardt D. S. Welz H. G. Geiger H. H. (1998b). Molecular marker analysis of European *Setosphaeria turcica* populations. Eur. J. Plant Pathol. 104, 611–617. doi: 10.1023/A:1008641920356

[B30] Bruns H. A. (2017). Southern corn leaf blight: a story worth retelling. Agron. J. 109, 1218–1224. doi: 10.2134/agronj2017.01.0006

[B31] Bubeck D. Goodman M. Beavis W. Grant D. (1993). Quantitative trait loci controlling resistance to gray leaf spot in maize. Crop Sci. 33, 838–847. doi: 10.2135/cropsci1993.0011183X003300040041x

[B32] Bunkoed W. Kasam S. Chaijuckam P. Yhamsoongnern J. Prathuangwong S. (2014). Sexual reproduction of *Setosphaeria turcica* in natural corn fields in Thailand. Agric. Natural Resour. 48 (2), 175–182.

[B33] Carbone I. Kohn L. M. (1999). A method for designing primer sets for speciation studies in filamentous ascomycetes. Mycologia 91 (3), 553–556. doi: 10.1080/00275514.1999.12061051

[B34] Carson M. Stuber C. Senior M. (2004). Identification and mapping of quantitative trait loci conditioning resistance to southern leaf blight of maize caused by *Cochliobolus heterostrophus* race O. Phytopathology 94, 862–867. doi: 10.1094/PHYTO.2004.94.8.862 18943107

[B35] Chaloner T. M. Gurr S. J. Bebber D. P. (2021). Plant pathogen infection risk tracks global crop yields under climate change. Nat. Climate Change 11, 710–715. doi: 10.1038/s41558-021-01104-8

[B36] Chang R.-Y. Peterson P. (1995). Genetic control of resistance to *Bipolaris maydis*: one gene or two genes? J. Heredity 86, 94–97. doi: 10.1093/oxfordjournals.jhered.a111555

[B37] Chapara V. Pedersen D. Balint-Kurti P. Esker P. Robertson A. Paul P.. (2012). Baseline sensitivity of *Exserohilum turcicum* to the quinone outside inhibitor pyraclostrobin. Phytopathology 102 (7), 21.

[B38] Chen G. Wang X. Long S. Jaqueth J. Li B. Yan J.. (2016). Mapping of QTL conferring resistance to northern corn leaf blight using high-density SNPs in maize. Mol. Breed. 36, 4. doi: 10.1007/s11032-015-0421-3

[B39] Chung C.-L. Jamann T. Longfellow J. Nelson R. (2010). Characterization and fine-mapping of a resistance locus for northern leaf blight in maize bin 8.06. Theor. Appl. Genet. 121, 205–227. doi: 10.1007/s00122-010-1303-z 20217383

[B40] Chung C.-L. Poland J. Kump K. Benson J. Longfellow J. Walsh E.. (2011). Targeted discovery of quantitative trait loci for resistance to northern leaf blight and other diseases of maize. Theor. Appl. Genet. 123, 307–326. doi: 10.1007/s00122-011-1585-9 21526397

[B41] Clements M. J. Dudley J. White D. (2000). Quantitative trait loci associated with resistance to gray leaf spot of corn. Phytopathology 90, 1018–1025. doi: 10.1094/PHYTO.2000.90.9.1018 18944528

[B42] Condon B. J. Elliott C. González J. B. Yun S. H. Akagi Y. Wiesner-Hanks T.. (2018). Clues to an evolutionary mystery: The genes for T-toxin, enabler of the devastating 1970 southern corn leaf blight epidemic, are present in ancestral species, suggesting an ancient origin. Mol. Plant-Microbe Interact. 31, 1154–1165. doi: 10.1094/MPMI-03-18-0070-R 29792566

[B43] Condon B. J. Leng Y. Wu D. Bushley K. E. Ohm R. A. Otillar R.. (2013). Comparative genome structure, secondary metabolite, and effector coding capacity across *Cochliobolus* pathogens. PloS Genet. 9, e1003233. doi: 10.1371/journal.pgen.1003233 23357949 PMC3554632

[B44] Craven M. Fourie A. (2011). Field evaluation of maize inbred lines for resistance to *Exserohilum turcicum* . South Afr. J. Plant Soil 28, 69–74. doi: 10.1080/02571862.2011.10640015

[B45] Craze H. A. Pillay N. Joubert F. Berger D. K. (2022). Deep learning diagnostics of gray leaf spot in maize under mixed disease field conditions. Plants 11, 1942. doi: 10.3390/plants11151942 35893646 PMC9330607

[B46] Crous P. W. Braun U. (2003). Mycosphaerella and its anamorphs: 1. Names published in Cercospora and Passalora (Centraalbureau voor Schimmelcultures (CBS), Fungal Biodiversity Centre, Uppsalalaan 8, 3584 CT Utrecht, Netherlands.

[B47] Crous P. W. Groenewald J. Z. Groenewald M. Caldwell P. Braun U. Harrington T. C. (2006). Species of *Cercospora* associated with grey leaf spot of maize. *Studies in Mycology* . 55 (1), 189–197. doi: 10.3114/sim.55.1.189 PMC2104713 18490979

[B48] Dai Y. Gan L. Ruan H. Shi N. Du Y. Liao L.. (2018). Sensitivity of *Cochliobolus heterostrophus* to three demethylation inhibitor fungicides, propiconazole, diniconazole and prochloraz, and their efficacy against southern corn leaf blight in Fujian Province, China. Eur. J. Plant Pathol. 152, 447–459. doi: 10.1007/s10658-018-1490-z

[B49] Danson J. Lagat M. Kimani M. Kuria A. (2008). Quantitative trait loci (QTLs) for resistance to gray leaf spot and common rust diseases of maize. Afr. J. Biotechnol. 7 (18), 3247–3254.

[B50] Dean J. Goodwin P. Hsiang T. (2005). Induction of glutathione S-transferase genes of *Nicotiana benthamiana* following infection by *Colletotrichum destructivum* and *C. orbiculare* and involvement of one in resistance. J. Exp. Bot. 56, 1525–1533. doi: 10.1093/jxb/eri145 15837710

[B51] DeChant C. Wiesner-Hanks T. Chen S. Stewart E. L. Yosinski J. Gore M. A.. (2017). Automated identification of northern leaf blight-infected maize plants from field imagery using deep learning. Phytopathology 107, 1426–1432. doi: 10.1094/PHYTO-11-16-0417-R 28653579

[B52] de Vallavieille-Pope C. Giosue S. Munk L. Newton A. Niks R. Østergård H.. (2000). Assessment of epidemiological parameters and their use in epidemiological and forecasting models of cereal airborne diseases. Agronomie EDP Sci. 20, 715–727. doi: 10.1051/agro:2000171

[B53] Dill-Macky R. Jones R. (2000). The effect of previous crop residues and tillage on *Fusarium* head blight of wheat. Plant Dis. 84, 71–76. doi: 10.1094/PDIS.2000.84.1.71 30841225

[B54] Dodd J. (2000). “How to foresee corn disease outbreaks,” in 55th Annual Corn & Sorghum Industry Research Conference, 55, 91–98.

[B55] Dong J. Fan Y. Gui X. An X. Ma J. Dong Z. (2008). Geographic distribution and genetic analysis of physiological races of *Setosphaeria turcica* in Northern China. Am. J. Agric. Biol. Sci. 3 (1), 389–398.

[B56] Drechsler C. (1923). Some graminicolons species of *Helminthosporium.* I. J. Agric. Res. 24 (8), 641–739.

[B57] Drechsler C. (1925). Leafspot of maize caused by Ophiobolus heterostrophus n. sp., the ascigerons stage of a *Helminthosporium* exhibiting bipolar germination. J. Agric. Res. 31 (8), 701726.

[B58] Duan C.-X. Zhao L.-P. Jie W. Liu Q.-K. Yang Z.-H. Wang X.-M. (2022). Dispersal routes of *Cercospora zeina* causing maize gray leaf spot in China. J. Integr. Agriculture 21 (10), 2943–2956. doi: 10.1016/j.jia.2022.07.042

[B59] Dunkle L. D. Levy M. (2000). Genetic relatedness of African and United States populations of *Cercospora zeae-maydis* . Phytopathology 90, 486–490. doi: 10.1094/PHYTO.2000.90.5.486 18944554

[B60] Duvick D. N. (2001). Biotechnology in the 1930s: the development of hybrid maize. Nat. Rev. Genet. 2, 69. doi: 10.1038/35047587 11253074

[B61] Dyer P. S. Ingram D. S. Johnstone K. (1992). The control of sexual morphogenesis in the *Ascomycotina* . Biol. Rev. 67, 421–458. doi: 10.1111/j.1469-185X.1992.tb01189.x

[B62] Dyer P. Paoletti M. (2005). Reproduction in Aspergillus fumigatus: sexuality in a supposedly asexual species? Med. Mycology 43 (S1), 7–14. doi: 10.1080/13693780400029015 16110786

[B63] Elad Y. Pertot I. (2014). Climate change impacts on plant pathogens and plant diseases. J. Crop Improvement 28, 99–139. doi: 10.1080/15427528.2014.865412

[B64] Ellwood S. R. Piscetek V. Mair W. J. Lawrence J. A. Lopez-Ruiz F. J. Rawlinson C. (2019). Genetic variation of *Pyrenophora teres* f. *teres* isolates in Western Australia and emergence of a Cyp51A fungicide resistance mutation. Plant Pathol. 68, 135–142. doi: 10.1111/ppa.12924

[B65] Emami K. Hack E. (2002). Conservation of *XYN11A* and *XYN11B* xylanase genes in *Bipolaris sorghicola*, *Cochliobolus sativus*, *Cochliobolus heterostrophus*, and *Cochliobolus spicifer* . Curr. Microbiol. 45, 303–306. doi: 10.1007/s00284-002-3618-8 12192531

[B66] Emechebe A. M. (1975). Some aspects of crop diseases in Uganda. CABI Database 19761329831:43.

[B67] Fan Y. Ma J. Gui X. An X. Sun S. Dong J. (2007). Distribution of mating types and genetic diversity induced by sexual recombination in *Setosphaeria turcica* in Northern China. Front. Agric. China 1, 368–376. doi: 10.1007/s11703-007-0062-3

[B68] FAOSTAT (2024). Food and Agriculture Organization, United Nations of Organization. Available online at: http://www.fao.org/faostat.

[B69] Ferguson L. M. Carson M. (2004). Spatial diversity of *Setosphaeria turcica* sampled from the Eastern United States. Phytopathology 94, 892–900. doi: 10.1094/PHYTO.2004.94.8.892 18943111

[B70] Ferguson L. Carson M. (2007). Temporal variation in Setosphaeria turcica between 1974 and 1994 and origin of races 1, 23, and 23N in the United States. Phytopathology 97, 1501–1511. doi: 10.1094/PHYTO-97-11-1501 18943521

[B71] Fisher M. C. Henk D. A. Briggs C. J. Brownstein J. S. Madoff L. C. McCraw S. L.. (2012). Emerging fungal threats to animal, plant and ecosystem health. Nature 484, 186. doi: 10.1038/nature10947 22498624 PMC3821985

[B72] Fisher D. Hooker A. Lim S. Smith D. (1976). Leaf infection and yield loss caused by four *Helminthosporium* leaf diseases of corn. Phytopathology 66, 942–944. doi: 10.1094/Phyto-66-942

[B73] Gafur A. Mujim S. Aeny T. N. (2002). Morphological and pathological variations in the Indonesian *Cochliobolus heterostrophus* (*Pleosporaceae*, *Pleosporales*, Euascomycetes). Pakistan J. Biol. Sci. 5, 1195–1198. doi: 10.3923/pjbs.2002.1195.1198

[B74] Gafur A. Tanaka C. Ouchi S. Tsuda M. (1997). A PCR-based method for mating type determination in *Cochliobolus heterostrophus* . Mycoscience 38, 455–458. doi: 10.1007/BF02461689

[B75] Gao Z. Xue Y. Dai J. (2000). The pathogenic site of the C-toxin derived from *Bipolaris maydis* race C in maize (*Zea mays*). Chin. Sci. Bull. 45, 1787–1791. doi: 10.1007/BF02886268

[B76] Gardes M. White T. J. Fortin J. A. Bruns T. D. Taylor J. W. (1991). Identification of indigenous and introduced symbiotic fungi in ectomycorrhizae by amplification of nuclear and mitochondrial ribosomal DNA. Can. J. Bot. 69, 180–190. doi: 10.1139/b91-026

[B77] Garnault M. Duplaix C. Leroux P. Couleaud G. Carpentier F. David O.. (2019). Spatiotemporal dynamics of fungicide resistance in the wheat pathogen *Zymoseptoria tritici* in France. Pest Manage. Sci. 75 (7), 1794–1807. doi: 10.1002/ps.5360 30680908

[B78] Gianasi L. Castro H. d. Silva H. d. (1996). Raças fisiológicas de *Exserohilum turcicum* identificadas em regiões produtoras de milho no Brasil, Safra 93/94. Summa Phytopathologica 22, 214–217.

[B79] Glass N. L. Donaldson G. C. (1995). Development of primer sets designed for use with the PCR to amplify conserved genes from filamentous ascomycetes. Appl. Environ. Microbiol. 61, 1323–1330. doi: 10.1128/aem.61.4.1323-1330.1995 7747954 PMC167388

[B80] Gogoi R. Singh S. Singh P. K. Kulanthaivel S. Rai S. (2014). Genetic variability in the isolates of *Bipolaris maydis* causing maydis leaf blight of maize. Afr. J. Agric. Res. 9, 1906–1913.

[B81] Goh T. Hyde K. Lee D. K. (1998). Generic distinction in the *Helminthosporium*-complex based on restriction analysis of the nuclear ribosomal RNA gene. Fungal Diversity 1, 85–107.

[B82] Gong M. Wang J. D. Zhang J. Yang H. LU X. F. Pei Y.. (2006). Study of the antifungal ability of *Bacillus subtilis* strain PY-1 *in vitro* and identification of its antifungal substance (iturin A). Acta Biochim. Biophys. Sin. 38, 233–240. doi: 10.1111/j.1745-7270.2006.00157.x 16604262

[B83] Goodwin S. B. Dunkle L. D. Zismann V. L. (2001). Phylogenetic analysis of *Cercospora* and *Mycosphaerella* based on the internal transcribed spacer region of ribosomal DNA. Phytopathology 91, 648–658. doi: 10.1094/PHYTO.2001.91.7.648 18942994

[B84] Gregory L. Ayers J. Nelson R. (1979). The influence of cultivar and location on yield loss in corn due to southern corn leaf blight [caused by *Helminthosporium maydis* race T]. Plant Dis. Rep. 63 (10), 891–895.

[B85] Groenewald M. Groenewald J. Z. Harrington T. C. Abeln E. C. Crous P. W. (2006). Mating type gene analysis in apparently asexual *Cercospora* species is suggestive of cryptic sex. Fungal Genet. Biol. 43, 813–825. doi: 10.1016/j.fgb.2006.05.008 16839791

[B86] Haasbroek M. Craven M. Barnes I. Crampton B. G. (2014). Microsatellite and mating type primers for the maize and sorghum pathogen, *Exserohilum turcicum* . Australas. Plant Pathol. 43, 577–581. doi: 10.1007/s13313-014-0289-4

[B87] Haridas S. González J. B. Riley R. Koriabine M. Yan M. Ng V.. (2023). T-toxin virulence genes: unconnected dots in a sea of repeats. MBio 14, e00261–e00223. doi: 10.1128/mbio.00261-23 36883814 PMC10128009

[B88] Henegariu O. Heerema N. Dlouhy S. Vance G. Vogt P. (1997). Multiplex PCR: critical parameters and step-by-step protocol. BioTechniques 23, 504–511. doi: 10.2144/97233rr01 9298224

[B89] Hernández-Restrepo M. Madrid H. Tan Y. Da Cunha K. Gene J. Guarro J.. (2018). Multi-locus phylogeny and taxonomy of *Exserohilum* . Persoonia: Mol. Phylogeny Evol. Fungi 41, 71. doi: 10.3767/persoonia.2018.41.05 PMC6344813 30728600

[B90] Hooda K. Khokhar M. Shekhar M. Karjagi C. G. Kumar B. Mallikarjuna N.. (2017). *Turcicum* leaf blight—sustainable management of a re-emerging maize disease. J. Plant Dis. Prot. 124, 101–113. doi: 10.1007/s41348-016-0054-8

[B91] Hou Y.-P. Chen Y.-L. Wu L.-Y. Wang J.-X. Chen C.-J. Zhou M.-G. (2018). Baseline sensitivity of *Bipolaris maydis* to the novel succinate dehydrogenase inhibitor benzovindiflupyr and its efficacy. Pesticide Biochem. Physiol. 149, 81–88. doi: 10.1016/j.pestbp.2018.06.002 30033021

[B92] Huff C. A. Ayers J. Hill R. Jr. (1988). Inheritance of resistance in corn (*Zea mays*) to gray leaf spot. Phytopathology 78, 790–794. doi: 10.1094/Phyto-78-790

[B93] Hulme P. E. (2009). Trade, transport and trouble: managing invasive species pathways in an era of globalization. J. Appl. Ecol. 46, 10–18. doi: 10.1111/j.1365-2664.2008.01600.x

[B94] Human M. P. Barnes I. Craven M. Crampton B. G. (2016). Lack of population structure and mixed reproduction modes in *Exserohilum turcicum* from South Africa. Phytopathology 106, 1386–1392. doi: 10.1094/PHYTO-12-15-0311-R 27392177

[B95] Human M. P. Berger D. K. Crampton B. G. (2020). Time-course RNAseq reveals *Exserohilum turcicum* effectors and pathogenicity determinants. Front. Microbiol. 11, 360. doi: 10.3389/fmicb.2020.00360 32265851 PMC7099616

[B96] Hurni S. Scheuermann D. Krattinger S. G. Kessel B. Wicker T. Herren G.. (2015). The maize disease resistance gene Htn1 against northern corn leaf blight encodes a wall-associated receptor-like kinase. Proc. Natl. Acad. Sci. 112, 8780–8785. doi: 10.1073/pnas.1502522112 26124097 PMC4507197

[B97] Inderbitzin P. Asvarak T. Turgeon B. G. (2010). Six new genes required for production of T-toxin, a polyketide determinant of high virulence of *Cochliobolus heterostrophus* to maize. Mol. Plant-Microbe Interact. 23, 458–472. doi: 10.1094/MPMI-23-4-0458 20192833

[B98] Jahani M. Aggarwal R. Srivastava K. (2011). Genetic differentiation of Bipolaris spp. based on random amplified polymorphic DNA markers. Indian Phytopathol. 61 (4), 449–455.

[B99] James T. Y. Kauff F. Schoch C. L. Matheny P. B. Hofstetter V. Cox C. J.. (2006). Reconstructing the early evolution of fungi using a six-gene phylogeny. Nature 443, 818. doi: 10.1038/nature05110 17051209

[B100] Jat H. Datta A. Choudhary M. Sharma P. C. Jat M. L. (2021). Conservation Agriculture: factors and drivers of adoption and scalable innovative practices in Indo-Gangetic plains of India–a review. Int. J. Agric. Sustainability 19, 40–55. doi: 10.1080/14735903.2020.1817655

[B101] Jeffers D. (2004). Maize diseases: a guide for field identification (Cimmyt), Mexio, D. F. Available online at: https://www.aflatoxinpartnership.org/wp-content/uploads/2021/05/MAIZE-DISEASES-PDF.pdf

[B102] Jindal K. K. Tenuta A. U. Woldemariam T. Zhu X. Hooker D. C. Reid L. M. (2019). Occurrence and distribution of physiological races of *Exserohilum turcicum* in Ontario, Canada. Plant Dis. 103, 1450–1457. doi: 10.1094/PDIS-06-18-0951-SR 31107641

[B103] Jordan E. G. Perkins J. M. Schall R. Pedersen W. (1983). Occurrence of race 2 of *Exserohilum turcicum* on corn in the central and eastern United States. Plant Dis. 67, 1163–1165. doi: 10.1094/PD-67-1163

[B104] Juliatti F. C. Pedrosa M. G. Silva H. D. da Silva J. V. C. (2009). Genetic mapping for resistance to gray leaf spot in maize. Euphytica 169, 227–238. doi: 10.1007/s10681-009-9943-2

[B105] Karimi M. R. (2003). Investigations on genetics of disease resistance on *Zea mays-Drechslera maydis* pathosystem and variability in *D. maydis*. Indian Agricultural Research Institute; New Delhi. http://krishikosh.egranth.ac.in/handle/1/5810008432

[B106] Karr A. L. Karr D. B. Strobel G. A. (1974). Isolation and partial characterization of four host-specific toxins of *Helminthosporium maydis* (race T). Plant Physiol. 53, 250–257. doi: 10.1104/pp.53.2.250 16658685 PMC541373

[B107] Kibe M. Nair S. K. Das B. Bright J. M. Makumbi D. Kinyua J.. (2020). Genetic dissection of resistance to gray leaf spot by combining genome-wide association, linkage mapping, and genomic prediction in tropical maize germplasm. Front. Plant Sci. 11, 572027. doi: 10.3389/fpls.2020.572027 33224163 PMC7667048

[B108] Kim P.-I. Ryu J.-W. Kim Y.-H. Chi Y.-T. (2010). Production of biosurfactant lipopeptides iturin A, fengycin, and surfactin A from *Bacillus subtilis* CMB32 for control of *Colletotrichum gloeosporioides* . J. Microbiol. Biotechnol. 20, 138–145. doi: 10.4014/jmb.0905.05007 20134245

[B109] Kinyua Z. Smith J. Kibata G. Simons S. Langat B. (2010). Status of grey leaf spot disease in Kenyan maize production ecosystems. Afr. Crop Sci. J. 18 (4), 183–194. doi: 10.4314/acsj.v18i4.68647

[B110] Knoema (2023). World: Maize production quantity. Available online at: https://knoema.com/atlas/World/topics/Agriculture/Crops-Production-Quantity-tonnes/Maize-production.

[B111] Korsman J. Meisel B. Kloppers F. J. Crampton B. G. Berger D. K. (2012). Quantitative phenotyping of grey leaf spot disease in maize using real-time PCR. Eur. J. Plant Pathol. 133, 461–471. doi: 10.1007/s10658-011-9920-1

[B112] Kotze R. van der Merwe C. Crampton B. Kritzinger Q. (2019). A histological assessment of the infection strategy of *Exserohilum turcicum* in maize. Plant Pathol. 68, 504–512. doi: 10.1111/ppa.12961

[B113] Kumar S. Pardurange Gowda K. Pant S. Shekhar M. Kumar B. Kaur B.. (2011). Sources of resistance to *Exserohilum turcicum* (Pass.) and *Puccinia polysora* (Underw.) incitant of *Turcicum* leaf blight and polysora rust of maize. Arch. Phytopathol. Plant Prot. 44, 528–536. doi: 10.1080/03235400903145558

[B114] Latterell F. M. Rossi A. E. (1983). Gray leaf spot of corn: a disease on the move. Plant Dis. 67, 842–847. doi: 10.1094/PD-67-842

[B115] Lee S. B. Taylor J. W. (1992). Phylogeny of five fungus-like protoctistan *Phytophthora* species, inferred from the internal transcribed spacers of ribosomal DNA. Mol. Biol. Evol. 9, 636–653. doi: 10.1093/oxfordjournals.molbev.a040750 1630304

[B116] Lehmensiek A. Esterhuizen A. Van Staden D. Nelson S. Retief A. (2001). Genetic mapping of gray leaf spot (GLS) resistance genes in maize. Theor. Appl. Genet. 103, 797–803. doi: 10.1007/s001220100599

[B117] Lennon J. R. Krakowsky M. Goodman M. Flint-Garcia S. Balint-Kurti P. J. (2016). Identification of alleles conferring resistance to gray leaf spot in maize derived from its wild progenitor species teosinte. Crop Sci. 56, 209–218. doi: 10.2135/cropsci2014.07.0468

[B118] Leonard K. (1974). *Bipolaris maydis* race and mating type frequencies in North Carolina. Plant Dis. Rep. 58, 529–531. https://www.cabidigitallibrary.org/doi/full/10.5555/19741314054

[B119] Leonard K. (1977a). Races of *Bipolaris maydis* in the southeastern US from 1974-1976. Plant Dis. Rep. 61, 914–915. https://www.cabidigitallibrary.org/doi/full/10.5555/19781664667

[B120] Leonard K. (1977b). Virulence, temperature optima, and competitive abilities of isolines of races T and O of *Bipolaris maydis* . Phytopathology 67 (11), 1273–1279. doi: 10.1094/Phyto-67-1273

[B121] Leonard K. Levy Y. Smith D. (1989). Proposed nomenclature for pathogenic races of *Exserohilum turcicum* on corn. Plant Dis. 73, 776–777. https://www.cabidigitallibrary.org/doi/full/10.5555/19901175159

[B122] Leonard K. Suggs E. G. (1974). *Setosphaeria prolata*, the ascigerous state of *Exserohilum prolatum* . Mycologia 66, 281–297. doi: 10.1080/00275514.1974.12019603

[B123] Levings C. S. (1990). The Texas cytoplasm of maize: cytoplasmic male sterility and disease susceptibility. Science 250, 942–947. doi: 10.1126/science.250.4983.942 17746917

[B124] Levings 3. C. (1993). Thoughts on cytoplasmic male sterility in cms-T maize. Plant Cell 5, 1285. doi: 10.1105/tpc.5.10.1285 12271028 PMC160361

[B125] Levy Y. Cohen Y. (1983). Biotic and environmental factors affecting infection of sweet corn with *Exserohilum turcicum* . Phytopathology 73, 722–725. doi: 10.1094/Phyto-73-722

[B126] Li Y.-x. Chen L. Li C. Bradbury P. J. Shi Y.-s. Song Y.. (2018). Increased experimental conditions and marker densities identified more genetic loci associated with southern and northern leaf blight resistance in maize. Sci. Rep. 8, 1–12. doi: 10.1038/s41598-018-25304-z 29717181 PMC5931595

[B127] Li Z. Liu Y. Hossain O. Paul R. Yao S. Wu S.. (2021). Real-time monitoring of plant stresses via chemiresistive profiling of leaf volatiles by a wearable sensor. Matter 4, 2553–2570. doi: 10.1016/j.matt.2021.06.009

[B128] Liang X. Zhang X. Xi K. Liu Y. Jijakli M. H. Guo W. (2024). Development of an RPA-based CRISPR/Cas12a assay in combination with a lateral flow strip for rapid detection of toxigenic *Fusarium verticillioides* in maize. Food Control 157, 110172. doi: 10.1016/j.foodcont.2023.110172

[B129] Lipps P. (1998). Gray leaf spot: a global threat to corn production (APSNet Feature). doi: 10.1094/APSnetFeature-1998-0598

[B130] Lipps P. White D. Ayers J. Dunkle L. (1998). “Gray leaf spot of corn: update,” in A report from NCR-25 technical committee on corn and sorghum diseases (The American Phytopathological Society). Available at: www.apsnet.org/online/feature/grayleaf/fullrprt.htm.

[B131] Liu K.-J. Xu X.-D. (2013). First report of gray leaf spot of maize caused by *Cercospora zeina* in China. Plant Dis. 97, 1656–1656. doi: 10.1094/PDIS-03-13-0280-PDN 30716816

[B132] Lopez-Zuniga L. O. Wolters P. Davis S. Weldekidan T. Kolkman J. M. Nelson R.. (2019). Using maize chromosome segment substitution line populations for the identification of loci associated with multiple disease resistance. G3: Genes Genomes Genet. 9, 189–201. doi: 10.1534/g3.118.200866 PMC6325898 30459178

[B133] Ma Z. Hui H. Huang Y. Yao Y. Sun Y. Liu B.. (2022). Evaluation of maize hybrids for identifying resistance to northern corn leaf blight in Northeast China. Plant Dis. 106, 1003–1008. doi: 10.1094/PDIS-09-21-1914-RE 34735284

[B134] Ma Z. Liu B. He S. Gao Z. (2020). Analysis of physiological races and genetic diversity of *Setosphaeria turcica* (Luttr.) KJ Leonard & Suggs from different regions of China. Can. J. Plant Pathol. 42, 396–407. doi: 10.1080/07060661.2019.1679261

[B135] Manamgoda D. S. Cai L. McKenzie E. H. Crous P. W. Madrid H. Chukeatirote E.. (2012). A phylogenetic and taxonomic re-evaluation of the *Bipolaris*-*Cochliobolus*-*Curvularia* complex. Fungal Diversity 56, 131–144. doi: 10.1007/s13225-012-0189-2

[B136] Manamgoda D. Rossman A. Y. Castlebury L. Crous P. W. Madrid H. Chukeatirote E.. (2014). The genus *Bipolaris* . Stud. Mycology 79, 221–288. doi: 10.1016/j.simyco.2014.10.002 PMC4255534 25492990

[B137] Manandhar G. Ferrara G. Tiwari T. Baidya S. Bajracharya A. Khadge B.. (2011). Response of maize genotypes to gray leaf spot disease (*Cercospora zeae-maydis*) in the hills of Nepal. Agron. J. Nepal 2, 93–101. doi: 10.3126/ajn.v2i0.7524

[B138] Manoj K. Agarwal V. K. (1998). Location of seedborne fungi associated with discoloured maize seeds. Indian Phytopathol. 51 (3), 247–250.

[B139] Manzar N. Kashyap A. S. Maurya A. Rajawat M. V. S. Sharma P. K. Srivastava A. K.. (2022). Multi-gene phylogenetic approach for identification and diversity analysis of *Bipolaris maydis* and *Curvularia lunata* isolates causing foliar blight of *Zea mays* . J. Fungi 8, 802. doi: 10.3390/jof8080802 PMC9410300 36012790

[B140] Marais I. Buitendag C. Duong T. A. Crampton B. G. Theron J. Kidanemariam D.. (2024). Double-stranded RNA uptake for the control of the maize pathogen *Cercospora zeina* . Plant Pathol. 73 (6) 1480–1490. doi: 10.1111/ppa.13909

[B141] Martins L. B. Rucker E. Thomason W. Wisser R. J. Holland J. B. Balint-Kurti P. (2019). Validation and characterization of maize multiple disease resistance QTL. G3: Genes Genomes Genet. 9, 2905–2912. doi: 10.1534/g3.119.400195 PMC6723135 31300480

[B142] McCartney H. A. Foster S. J. Fraaije B. A. Ward E. (2003). Molecular diagnostics for fungal plant pathogens. Pest Manage. Sci. 59, 129–142. doi: 10.1002/ps.575 12587866

[B143] McDonald B. A. (1997). The population genetics of fungi: tools and techniques. Phytopathology 87, 448–453. doi: 10.1094/PHYTO.1997.87.4.448 18945126

[B144] McDonald B. A. Linde C. (2002). Pathogen population genetics, evolutionary potential, and durable resistance. Annu. Rev. Phytopathol. 40, 349–379. doi: 10.1146/annurev.phyto.40.120501.101443 12147764

[B145] Meisel B. Korsman J. Kloppers F. J. Berger D. K. (2009). *Cercospora zeina* is the causal agent of grey leaf spot disease of maize in southern Africa. Eur. J. Plant Pathol. 124, 577–583. doi: 10.1007/s10658-009-9443-1

[B146] Milgroom M. G. Peever T. L. (2003). Population biology of plant pathogens: the synthesis of plant disease epidemiology and population genetics. Plant Dis. 87, 608–617. doi: 10.1094/PDIS.2003.87.6.608 30812848

[B147] Miller S. A. Beed F. D. Harmon C. L. (2009). Plant disease diagnostic capabilities and networks. Annu. Rev. Phytopathol. 47, 15–38. doi: 10.1146/annurev-phyto-080508-081743 19385729

[B148] Moghaddam P. F. Pataky J. (1994). Reactions of isolates from matings of races 1 and 23N of *Exserohilum turcicum* . Plant Dis. 767, 767–771. doi: 10.1094/PD-78-0767

[B149] Mohanty S. P. Hughes D. P. Salathé M. (2016). Using deep learning for image-based plant disease detection. Front. Plant Sci. 7, 1419. doi: 10.3389/fpls.2016.01419 27713752 PMC5032846

[B150] Mueller D. S. Wise K. A. Sisson A. J. Allen T. W. Bergstrom G. C. Bissonnette K. M.. (2020). Corn yield loss estimates due to diseases in the United States and Ontario, Canada, from 2016 to 2019. Plant Health Prog. 21, 238–247. doi: 10.1094/PHP-05-20-0038-RS

[B151] Mueller D. S. Wise K. A. Sisson A. J. Allen T. W. Bergstrom G. C. Bosley D. B.. (2016). Corn yield loss estimates due to diseases in the United States and Ontario, Canada from 2012 to 2015. Plant Health Prog. 17, 211–222. doi: 10.1094/PHP-RS-16-0030

[B152] Muiru W. Koopmann B. Tiedemann A. Mutitu E. Kimenju J. (2010). Race typing and evaluation of aggressiveness of *Exserohilum turcicum* isolates of Kenyan, German and Austrian origin. World J. Agric. Sci. 6 (3), 277–284.

[B153] MuLaosmanovic E. Lindblom T. U. Bengtsson M. Windstam S. T. Mogren L. Marttila S.. (2020). High-throughput method for detection and quantification of lesions on leaf scale based on trypan blue staining and digital image analysis. Plant Methods 16, 1–22. doi: 10.1186/s13007-020-00605-5 32391069 PMC7197134

[B154] Muller M. F. Barnes I. Kunene N. T. Crampton B. G. Bluhm B. H. Phillips S. M.. (2016). *Cercospora zeina* from maize in South Africa exhibits high genetic diversity and lack of regional population differentiation. Phytopathology 106, 1194–1205. doi: 10.1094/PHYTO-02-16-0084-FI 27392176

[B155] Munjal E. Kapoor J. (1960). Some unrecorded diseases of sorghum and maize from India. Curr. Sci. 29 (11), 442–443.

[B156] Munkvold G. Martinson C. Shriver J. Dixon P. (2001). Probabilities for profitable fungicide use against gray leaf spot in hybrid maize. Phytopathology 91, 477–484. doi: 10.1094/PHYTO.2001.91.5.477 18943592

[B157] Muñoz-Zavala C. Loladze A. Vargas-Hernández M. García-León E. Alakonya A. E. Tovar-Pedraza J. M.. (2023). Occurrence and distribution of physiological races of *Exserohilum turcicum* in maize-growing regions of Mexico. Plant Dis. 107, 1054–1059. doi: 10.1094/PDIS-03-22-0626-RE 36089680

[B158] Mutka A. M. Bart R. S. (2015). Image-based phenotyping of plant disease symptoms. Front. Plant Sci. 5, 734. doi: 10.3389/fpls.2014.00734 25601871 PMC4283508

[B159] Mwangi S. F. M. (1998). *Status of northern leaf blight, Phaeosphaeria maydis leaf spot, Southern leaf blight, rust, maize streak virus and physiologic specialization of Exserohilum turcicum in Kenya* . Blacksburg, Virginia, USA: Virginia Polytechnic Institute and State University. Doctoral Dissertation.

[B160] Navarro B. L. Ramos Romero L. Kistner M. B. Iglesias J. Von Tiedemann A. (2021). Assessment of physiological races of *Exserohilum turcicum* isolates from maize in Argentina and Brazil. Trop. Plant Pathol. 46, 371–380. doi: 10.1007/s40858-020-00417-x

[B161] Negeri A. T. Coles N. D. Holland J. B. Balint-Kurti P. J. (2011). Mapping QTL controlling southern leaf blight resistance by joint analysis of three related recombinant inbred line populations. Crop Sci. 51, 1571–1579. doi: 10.2135/cropsci2010.12.0672

[B162] Nelson R. (1960). Evolution of sexuality and pathogenicity. 1. Interspecific crosses in the genus *Helminthosporium* . Phytopathology 50, 375–377.

[B163] Nelson R. Wiesner-Hanks T. Wisser R. Balint-Kurti P. (2018). Navigating complexity to breed disease-resistant crops. Nat. Rev. Genet. 19, 21. doi: 10.1038/nrg.2017.82 29109524

[B164] Neves D. L. Bradley C. A. (2019). Baseline sensitivity of *Cercospora zeae-maydis* to pydiflumetofen, a new succinate dehydrogenase inhibitor fungicide. Crop Prot. 119, 177–179. doi: 10.1016/j.cropro.2019.01.021

[B165] Neves D. L. Silva C. N. Pereira C. B. Campos H. D. Tessmann D. J. (2015). *Cercospora zeina* is the main species causing gray leaf spot in southern and central Brazilian maize regions. Trop. Plant Pathol. 40, 368–374. doi: 10.1007/s40858-015-0053-5

[B166] Nieuwoudt A. Human M. Craven M. Crampton B. (2018). Genetic differentiation in populations of *Exserohilum turcicum* from maize and sorghum in South Africa. Plant Pathol. 67, 1483–1491. doi: 10.1111/ppa.12858

[B167] Nsibo D. L. Barnes I. Kunene N. T. Berger D. K. (2019). Influence of farming practices on the population genetics of the maize pathogen *Cercospora zeina* in South Africa. Fungal Genet. Biol. 125, 36–44. doi: 10.1016/j.fgb.2019.01.005 30659907

[B168] Nsibo D. L. Barnes I. Omondi D. O. Dida M. M. Berger D. K. (2021). Population genetic structure and migration patterns of the maize pathogenic fungus, *Cercospora zeina* in East and Southern Africa. Fungal Genet. Biol. 149, 103527. doi: 10.1016/j.fgb.2021.103527 33524555

[B169] Nutter F. Jr. Gleason M. Jenco J. Christians N. (1993). Assessing the accuracy, intra-rater repeatability, and inter-rater reliability of disease assessment systems. Phytopathology 83, 806–812. doi: 10.1094/Phyto-83-806

[B170] Nwanosike M. Mabagala R. Kusolwa P. (2015). Effect of northern leaf blight (*Exserohilum turcicum*) severity on yield of maize (*Zea mays* L.) in Morogoro, Tanzania. Int. J. Sci. Res. 4 (9), 465–474.

[B171] Ogliari J. B. Guimarães M. A. Geraldi I. O. Camargo L. E. A. (2005). New resistance genes in the *Zea mays: Exserohilum turcicum* pathosystem. Genet. Mol. Biol. 28, 435–439. doi: 10.1590/S1415-47572005000300017

[B172] Ohm R. A. Feau N. Henrissat B. Schoch C. L. Horwitz B. A. Barry K. W.. (2012). Diverse lifestyles and strategies of plant pathogenesis encoded in the genomes of eighteen Dothideomycetes fungi. PloS Pathog. 8, e1003037. doi: 10.1371/journal.ppat.1003037 23236275 PMC3516569

[B173] Okori P. Fahleson J. Rubaihayo P. Adipala E. Dixelius C. (2003). Assessment of genetic variation among East African *Cercospora zeae-maydis* . J. Afr. Crop Sci. 11 (1), 75–85. doi: 10.4314/acsj.v11i2.27520

[B174] Okori P. Rubaihayo P. Adipala E. Fahieson J. Dixelius C. (2015). Dynamics of *Cercospora zeina* populations in maize-based agro-ecologies of Uganda. J. Afr. Crop Sci. 23, 45–57.

[B175] Omondi D. O. Dida M. M. Berger D. K. Beyene Y. Nsibo D. L. Juma C.. (2023). Combination of linkage and association mapping with genomic prediction to infer QTL regions associated with gray leaf spot and northern corn leaf blight resistance in tropical maize. Front. Genet. 14, 1282673. doi: 10.3389/fgene.2023.1282673 38028598 PMC10661943

[B176] Pal I. Singh V. Gogoi R. Hooda K. Bedi N. (2015). Characterization of *Bipolaris maydis* isolates of different maize cropping zones of India. Indian Phytopathol. 68 (1), 63–66.

[B177] Pan S.-Q. Qiao J.-F. Rui W. Yu H.-L. Cheng W. Taylor K.. (2022). Intelligent diagnosis of northern corn leaf blight with deep learning model. J. Integr. Agric. 21, 1094–1105. doi: 10.1016/S2095-3119(21)63707-3

[B178] Paul P. Munkvold G. (2005). Influence of temperature and relative humidity on sporulation of *Cercospora zeae-maydis* and expansion of gray leaf spot lesions on maize leaves. Plant Dis. 89, 624–630. doi: 10.1094/PD-89-0624 30795388

[B179] Pauli D. Chapman S. C. Bart R. Topp C. N. Lawrence-Dill C. J. Poland J.. (2016). The quest for understanding phenotypic variation via integrated approaches in the field environment. Plant Physiol. 172, 622–634. doi: 10.1104/pp.16.00592 27482076 PMC5047081

[B180] Pavan G. Shete P. (2021). Symptomatology, etiology, epidemiology and management of Southern corn leaf blight of maize (*Bipolaris maydis*)(Nisikado and Miyake) Shoemaker. Pharma Innovation J. 10 (5), 840–844.

[B181] Payne G. Waldron J. (1983). Overwintering and spore release of *Cercospora zeae-maydis* in corn debris in North Carolina. Plant Dis. 67, 87–89. doi: 10.1094/PD-67-87

[B182] Poland J. A. Balint-Kurti P. J. Wisser R. J. Pratt R. C. Nelson R. J. (2009). Shades of gray: the world of quantitative disease resistance. Trends Plant Sci. 14, 21–29. doi: 10.1016/j.tplants.2008.10.006 19062327

[B183] Poland J. A. Bradbury P. J. Buckler E. S. Nelson R. J. (2011). Genome-wide nested association mapping of quantitative resistance to northern leaf blight in maize. Proc. Natl. Acad. Sci. 108, 6893–6898. doi: 10.1073/pnas.1010894108 21482771 PMC3084105

[B184] Poland J. A. Nelson R. J. (2011). In the eye of the beholder: the effect of rater variability and different rating scales on QTL mapping. Phytopathology 101, 290–298. doi: 10.1094/PHYTO-03-10-0087 20955083

[B185] Prakash A. Rao J. Mukherjee A. K. Berliner J. Pokhare S. S. Adak T.. (2014). Climate change: impact on crop pests (Applied Zoologists Research Association (AZRA), Central Rice Research Institute). Available at: https://www.researchgate.net/profile/Berliner-Jeyaveeran/publication/275347570_Climate_Change_Impact_on_Crop_Pests/links/5593758508ae5af2b0eb7fd3/Climate-Change-Impact-on-Crop-Pests.pdf.

[B186] Pryce T. M. Palladino S. Kay I. Coombs G. (2003). Rapid identification of fungi by sequencing the ITS1 and ITS2 regions using an automated capillary electrophoresis system. Med. mycology 41, 369–381. doi: 10.1080/13693780310001600435 14653513

[B187] Qi Z. Jiang Z. Yang C. Liu L. Rao Y. (2016). Identification of maize leaf diseases based on image technology. J. Anhui Agric. Univ. 43 (2), 325–330.

[B188] Ramathani I. Biruma M. Martin T. Dixelius C. Okori P. (2011). Disease severity, incidence and races of *Setosphaeria turcica* on sorghum in Uganda. Eur. J. Plant Pathol. 131, 383–392. doi: 10.1007/s10658-011-9815-1

[B189] Rashid Z. Sofi M. Harlapur S. I. Kachapur R. M. Dar Z. A. Singh P. K.. (2020). Genome-wide association studies in tropical maize germplasm reveal novel and known genomic regions for resistance to northern corn leaf blight. Sci. Rep. 10, 1–16. doi: 10.1038/s41598-020-78928-5 33319847 PMC7738672

[B190] Ray D. K. Mueller N. D. West P. C. Foley J. A. (2013). Yield trends are insufficient to double global crop production by 2050. PloS One 8, e66428. doi: 10.1371/journal.pone.0066428 23840465 PMC3686737

[B191] Reddy T. R. Reddy P. N. Reddy R. R. Reddy S. S. (2013). Management of Turcicum leaf blight of maize caused by *Exserohilum turcicum* in maize. Int. J. Sci. Res. Publications 3, 1–4. http://www.ijsrp.org/e-journal.html

[B192] Reicosky D. C. (2021). “Carbon management in conservation agriculture systems,” in Regenerative Agriculture (Springer), 33–45.

[B193] Robert A. L. (1953). “Some of the leaf blights of corn,” in Year Book of Agriculture, CABI Databases 19541601980, 380–385.

[B194] Robeson D. Strobel G. (1982). Monocerin, a phytotoxin from *Exserohilum turcicum* (*Drechslera turcica*). Agric. Biol. Chem. 46, 2681–2683. doi: 10.1080/00021369.1982.10865494

[B195] Rodenburg J. Büchi L. Haggar J. (2020). Adoption by adaptation: Moving from conservation agriculture to conservation practices. Int. J. Agric. Sustainability 19 (5-6), 437–455. doi: 10.1080/14735903.2020.1785734

[B196] Rong I. H. Baxter A. P. (2006). The South African national collection of fungi: celebrating a centenary 1905-2005. Stud. Mycology 55, 1–12. doi: 10.3114/sim.55.1.1 PMC2104721 18490968

[B197] Rossman A. Y. Manamgoda D. S. Hyde K. D. (2013). (2233) Proposal to conserve the name *Bipolaris* against *Cochliobolus* (Ascomycota: Pleosporales: Pleosporaceae). Taxon 62, 1331–1332. doi: 10.12705/626.21

[B198] Savary S. Willocquet L. Pethybridge S. J. Esker P. McRoberts N. Nelson A. (2019). The global burden of pathogens and pests on major food crops. Nat. Ecol. Evol. 3 (3), 430–439. doi: 10.1038/s41559-018-0793-y 30718852

[B199] Schoch C. L. Seifert K. A. Huhndorf S. Robert V. Spouge J. L. Levesque C. A.. (2012). Nuclear ribosomal internal transcribed spacer (ITS) region as a universal DNA barcode marker for fungi. Proc. Natl. Acad. Sci. 109, 6241–6246. doi: 10.1073/pnas.1117018109 22454494 PMC3341068

[B200] Schwartz H. F. David G. H. (2005). “Sweet Corn VI, *Helminthosporium* leaf blight,” in High Plains IPM Guide, a cooperative effort of the University of Wyoming, University of Nebraska, Colorado State University and Montana State University. Available at: https://www.cabdirect.org/cabdirect/FullTextPDF/2014/20143334606.pdf.

[B201] Shah D. Dillard H. (2010). Managing foliar diseases of processing sweet corn in New York with strobilurin fungicides. Plant Dis. 94, 213–220. doi: 10.1094/PDIS-94-2-0213 30754265

[B202] Sharma R. Payak M. (1990). Durable resistance to two leaf blights in two maize inbred lines. Theor. Appl. Genet. 80, 542–544. doi: 10.1007/BF00226757 24221014

[B203] Sharma P. Sharma S. (2016). Paradigm shift in plant disease diagnostics: a journey from conventional diagnostics to nano-diagnostics. Curr. Trends Plant Dis. Diagnostics Manage. Practices Fungal Biology. Cham: Springer. doi: 10.1007/978-3-319-27312-9_11

[B204] Shi N. Du Y. Ruan H. Yang X. Dai Y. Gan L.. (2017). First report of northern corn leaf blight caused by *Setosphaeria turcica* on Corn (*Zea mays*) in Fujian Province, China. Plant Dis. 101, 831. doi: 10.1094/PDIS-07-16-0942-PDN

[B205] Simmons C. R. Grant S. Altier D. J. Dowd P. F. Crasta O. Folkerts O.. (2001). Maize *rhm1* resistance to *Bipolaris maydis* is associated with few differences in pathogenesis-related proteins and global mRNA profiles. Mol. Plant-Microbe Interact. 14, 947–954. doi: 10.1094/MPMI.2001.14.8.947 11497466

[B206] Simón M. R. Ayala F. M. Golik S. I. Terrile I. I. Cordo C. A. Perelló A. E.. (2011). Integrated foliar disease management to prevent yield loss in Argentinian wheat production. Agron. J. 103, 1441–1451. doi: 10.2134/agronj2010.0513

[B207] Singh A. Ganapathysubramanian B. Singh A. K. Sarkar S. (2016). Machine learning for high-throughput stress phenotyping in plants. Trends Plant Sci. 21, 110–124. doi: 10.1016/j.tplants.2015.10.015 26651918

[B208] Singh R. P. Hodson D. P. Jin Y. Huerta-Espino J. Kinyua M. G. Wanyera R.. (2006). Current status, likely migration and strategies to mitigate the threat to wheat production from race Ug99 (TTKS) of stem rust pathogen. Cab Reviews: Perspect. Agriculture Veterinary Science Nutr. Natural Resour. 1 (54), 1–13. doi: 10.1079/PAVSNNR200610

[B209] Singh R. Srivastava R. (2012). Southern corn leaf blight-an important disease of maize: an extension fact sheet. Indian Res. J. Extension Educ. 12 (2), 324–327.

[B210] Smith D. Hooker A. Lim S. (1970). Physiologic races of *Helminthosporium maydis* . Plant Dis. Rep. 54, 819–822.

[B211] Sperschneider J. Dodds P. N. (2022). EffectorP 3.0: prediction of apoplastic and cytoplasmic effectors in fungi and oomycetes. Mol. Plant Microbe Interact. 35, 146–156. doi: 10.1094/MPMI-08-21-0201-R 34698534

[B212] St. Clair D. A. (2010). Quantitative disease resistance and quantitative resistance loci in breeding. Annu. Rev. Phytopathol. 48, 247–268. doi: 10.1146/annurev-phyto-080508-081904 19400646

[B213] Stewart E. L. McDonald B. A. (2014). Measuring quantitative virulence in the wheat pathogen *Zymoseptoria tritici* using high-throughput automated image analysis. Phytopathology 104, 985–992. doi: 10.1094/PHYTO-11-13-0328-R 24624955

[B214] Sun S-q. Wen L-l. Dong J-g. (2005). Identification of physiological races and mating type of *Exserohilum turcicum* . Journal of Maize Sciences 13, 112–113.

[B215] Sun J. Pang C. Cheng X. Yang B. Jin B. Jin L.. (2023). Investigation of the antifungal activity of the dicarboximide fungicide iprodione against *Bipolaris maydis*. Pesticide Biochem. Physiol. 190(105319), 1–11. doi: 10.1016/j.pestbp.2022.105319 36740339

[B216] Swart V. Crampton B. G. Ridenour J. B. Bluhm B. H. Olivier N. A. Meyer J. M.. (2017). Complementation of *CTB7* in the maize pathogen *Cercospora zeina* overcomes the lack of *in vitro* cercosporin production. Mol. Plant-Microbe Interact. 30, 710–724. doi: 10.1094/MPMI-03-17-0054-R 28535078

[B217] Tan Y. P. Crous P. W. Shivas R. G. (2016). Eight novel *Bipolaris* species identified from John L. Alcorn’s collections at the Queensland Plant Pathology Herbarium (BRIP). Mycological Prog. 15, 1203–1214. doi: 10.1007/s11557-016-1240-6

[B218] Tang L. Gao Z. Yao Y. Liu X. (2015). Identification and genetic diversity of formae speciales of *Setosphaeria turcica* in China. Plant Dis. 99, 482–487. doi: 10.1094/PDIS-06-14-0570-RE 30699554

[B219] Technow F. Bürger A. Melchinger A. E. (2013). Genomic prediction of northern corn leaf blight resistance in maize with combined or separated training sets for heterotic groups. G3: Genes| Genomes| Genet. 3, 197–203. doi: 10.1534/g3.112.004630 PMC3564980 23390596

[B220] Tehon R. Daniels E. (1925). Notes on the parasitic fungi of Illinois-II. Mycologia 17, 240–249. doi: 10.1080/00275514.1925.12020479

[B221] Tilahun T. Wagary D. Demissie G. Negash M. Admassu S. Jifar H. (2012). “Maize pathology research in Ethiopia in the 2000s: A review,” in Meeting the Challenges of Global Climate Change and Food Security through Innovative Maize Research, 193.

[B222] Turgay E. B. Büyük O. Tunalı B. Helvacıoğlu Ö. Kurt Ş. (2020). Detection of the race of *Exserohilum turcicum* [(Pass.) KJ Leonard & Suggs] causing northern leaf blight diseases of corn in Turkey. J. Plant Pathol. 102, 387–393. doi: 10.1007/s42161-019-00440-1

[B223] Turgay E. B. Çelik Oğuz A. Ölmez F. Tunali B. Kurt Ş. Akçali E.. (2021). Genetic diversity and mating-type frequency of *Exserohilum turcicum* in Turkey. J. Phytopathol. 169, 570–576. doi: 10.1111/jph.13029

[B224] Turgeon B. G. Sharon A. Wirsel S. Yamaguchi K. Christiansen S. K. Yoder O. C. (1995). Structure and function of mating type genes in Cochliobolus spp. and asexual fungi. Can. J. Bot. 73, 778–783. doi: 10.1139/b95-322

[B225] Ullstrup A. (1972). The impacts of the southern corn leaf blight epidemics of 1970-1971. Annu. Rev. Phytopathol. 10, 37–50. doi: 10.1146/annurev.py.10.090172.000345

[B226] Van Staden D. Lambert C. Lehmensiek A. (2001). SCAR markers for the *Ht1*, *Ht2*, *Ht3* and *HtN1* resistance genes in maize. Maize Genet. Conf. Abstract 43, 134.

[B227] Vivek B. S. Odongo O. Njuguna J. Imanywoha J. Bigirwa G. Pixley K. (2010). Diallel analysis of grain yield and resistance to seven diseases of 12 African maize (*Zea mays* L.) inbred lines. Euphytica 172, 329–340. doi: 10.1007/s10681-009-9993-5

[B228] Vleeshouwers V. G. A. A. Oliver R. P. (2014). Effectors as tools in disease resistance breeding against biotrophic, hemibiotrophic, and necrotrophic plant pathogens. Mol. Plant-Microbe Interact. 27, 196–206. doi: 10.1094/MPMI-10-13-0313-IA 24405032

[B229] Walker D. M. Castlebury L. A. Rossman A. Y. White J. F. Jr (2012). New molecular markers for fungal phylogenetics: two genes for species-level systematics in the Sordariomycetes (Ascomycota). Mol. Phylogenet. Evol. 64, 500–512. doi: 10.1016/j.ympev.2012.05.005 22626621

[B230] Wang L. Kang Z. Wu Y. Zhou H. Mao Z. He Y. (2010). Preliminary identification of physiological races of *Bipolaris maydis* in Yunnan. J. Yunnan University-Natural Sci. Edition 32, 352–357.

[B231] Wang J. Levy M. Dunkle L. D. (1998). Sibling species of *Cercospora* associated with gray leaf spot of maize. Phytopathology 88, 1269–1275. doi: 10.1094/PHYTO.1998.88.12.1269 18944828

[B232] Wang J. Xu Z. Yang J. Lu X. Zhou Z. Zhang C.. (2018). qNCLB7. 02, a novel QTL for resistance to northern corn leaf blight in maize. Mol. Breed. 38 (5), 54. doi: 10.1007/s11032-017-0770-1

[B233] Ward E. Foster S. J. Fraaije B. A. McCartney H. A. (2004). Plant pathogen diagnostics: immunological and nucleic acid-based approaches. Ann. Appl. Biol. 145, 1–16. doi: 10.1111/j.1744-7348.2004.tb00354.x

[B234] Ward J. Laing M. Nowell D. (1997). Chemical control of maize grey leaf spot. Crop Prot. 16, 265–271. doi: 10.1016/S0261-2194(96)00097-X

[B235] Ward J. M. Nowell D. (1998). Integrated management practices for the control of maize grey leaf spot. Integrated Pest Manage. Rev. 3, 177–188. doi: 10.1023/A:1009694632036

[B236] Ward J. M. Stromberg E. L. Nowell D. C. Nutter F. W. Jr. (1999). Gray leaf spot: a disease of global importance in maize production. Plant Dis. 83, 884–895. doi: 10.1094/PDIS.1999.83.10.884 30841068

[B237] Warren H. (1975). Temperature effects on lesion development and sporulation after infection by races O and T of *Bipolaris maydis* . Phytopathology 65, 623–626. doi: 10.1094/Phyto-65-623

[B238] Weems J. D. Bradley C. A. (2017). Sensitivity of *Exserohilum turcicum* to demethylation inhibitor fungicides. Crop Prot. 99, 85–92. doi: 10.1016/j.cropro.2017.05.011

[B239] Weems J. D. Bradley C. A. (2018). *Exserohilum turcicum* race population distribution in the North-Central United States. Plant Dis. 102, 292–299. doi: 10.1094/PDIS-01-17-0128-RE 30673529

[B240] Wei J.-K. Liu K.-M. Chen J.-P. Luo P.-C. Stadelmann O. (1988). Pathological and physiological identification of race C of *Bipolaris maydis* in China. Phytopathology 78, 550–554. doi: 10.1094/Phyto-78-550

[B241] Weikert-Oliveira R. C. Resende M. Valério H. M. Caligiorne R. B. Paiva E. (2002). Genetic variation among pathogens causing" Helminthosporium" diseases of rice, maize and wheat. Fitopatologia Bras. 27, 639–643. doi: 10.1590/S0100-41582002000600015

[B242] Welgemoed T. Duong T. A. Barnes I. Stukenbrock E. H. Berger D. K. (2023). Population genomic analyses suggest recent dispersal events of the pathogen *Cercospora zeina* into East and Southern African maize cropping systems. G3: Genes Genomes Genet. 13 (11), 1–16, jkad214. doi: 10.1093/g3journal/jkad214 PMC10627275 37738420

[B243] Welz H. Geiger H. (2000). Genes for resistance to northern corn leaf blight in diverse maize populations. Plant Breed. 119 (1), 1–14. doi: 10.1046/j.1439-0523.2000.00462.x

[B244] Welz H. Wagner R. Geiger H. (1993). Virulence variation in *Setosphaeria turcica* populations collected from maize in China, Mexico, Uganda, and Zambia. Phytopathology 83, 1356.

[B245] Wende A. Shimelis H. Gwata E. T. (2018). Genetic variability for resistance to leaf blight and diversity among selected maize inbred lines (IntechOpen Limited). doi: 10.5772/intechopen.70553

[B246] Wheatley M. S. Duan Y.-P. Yang Y. (2021). Highly sensitive and rapid detection of citrus Huanglongbing pathogen (‘*Candidatus* Liberibacter asiaticus’) using Cas12a-based methods. Phytopathology® 111, 2375–2382. doi: 10.1094/PHYTO-09-20-0443-R 33944602

[B247] White D. G. (1999). Compendium of corn diseases Vol. 78 (APS press St. Paul, MN).

[B248] White J. Calvert O. Brown M. (1973). Ultrastructure of the conidia of *Helminthosporium maydis* . Can. J. Bot. 51, 2006–2008. doi: 10.1139/b73-261

[B249] Windes J. Pederson W. (1991). An isolate of *Exserohilum turcicum* virulent on maize inbreds with resistance gene *HtN* . Plant Disease, vol. 75, 430–430.

[B250] Wiesner-Hanks T. Nelson R. (2016). Multiple disease resistance in plants. Annual Review of Phytopathology 54 (1), 229–252. 10.1146/annurev-phyto-080615-100037 27296142

[B251] Wingfield B. D. Berger D. K. Steenkamp E. T. Lim H.-J. Duong T. A. Bluhm B. H.. (2017). Draft genome of Cercospora zeina, Fusarium *pininemorale*, *Hawksworthiomyces lignivorus*, *Huntiella decipiens* and *Ophiostoma ips* . IMA Fungus 8 (2), 385–396. doi: 10.5598/imafungus.2017.08.02.10 29242781 PMC5729718

[B252] Wingfield B.D. Berger D.K. Coetzee M. P.A. Duong T.A. Martin A. Pham N.Q.. (2022). IMA genome-F17 Draft genome sequences of an *Armillaria* species from Zimbabwe, *Ceratocystis colombiana*, *Elsinoë necatrix*, *Rosellinia necatrix*, two genomes of *Sclerotinia minor*, short-read genome assemblies and annotations of four *Pyrenophora teres* isolates from barley grass, and a long-read genome assembly of *Cercospora zeina* . IMA Fungus 13 (19), 122. 10.1186/s43008-022-00104-3 PMC9677705 36411457

[B253] Wisser R. J. Balint-Kurti P. J. Nelson R. J. (2006). The genetic architecture of disease resistance in maize: a synthesis of published studies. Phytopathology 96, 120–129. doi: 10.1094/PHYTO-96-0120 18943914

[B254] Wisser R. J. Kolkman J. M. Patzoldt M. E. Holland J. B. Yu J. Krakowsky M.. (2022). Multivariate analysis of maize disease resistances suggests a pleiotropic genetic basis and implicates a GST gene. Proc. Natl. Acad. Sci. 108, 7339–7344. doi: 10.1073/pnas.1011739108 PMC3088610 21490302

[B255] Worku M. De Groote H. Munyua B. Makumbi D. Owino F. Crossa J.. (2020). On-farm performance and farmers’ participatory assessment of new stress-tolerant maize hybrids in Eastern Africa. Field Crops Res. 246, 107693. doi: 10.1016/j.fcr.2019.107693 32015590 PMC6961973

[B256] Xie W. Yu K. Pauls K. P. Navabi A. (2012). Application of image analysis in studies of quantitative disease resistance, exemplified using common bacterial blight–common bean pathosystem. Phytopathology 102, 434–442. doi: 10.1094/PHYTO-06-11-0175 22204655

[B257] Xu Y. Crouch J. H. (2008). Marker-assisted selection in plant breeding: from publications to practice. Crop Sci. 48, 391–407. doi: 10.2135/cropsci2007.04.0191

[B258] Xu L. Xu X. Hu M. Wang R. Xie C. Chen H. (2015). Corn leaf disease identification based on multiple classifiers fusion. Trans. Chin. Soc. Agric. Eng. 31 (14), 194–201.

[B259] Yang Q. He Y. Kabahuma M. Chaya T. Kelly A. Borrego E.. (2017). A gene encoding maize caffeoyl-CoA O-methyltransferase confers quantitative resistance to multiple pathogens. Nat. Genet. 49, 1364–1372. doi: 10.1038/ng.3919 28740263

[B260] Yang P. Scheuermann D. Kessel B. Koller T. Greenwood J. R. Hurni S.. (2021). Alleles of a wall-associated kinase gene account for three of the major northern corn leaf blight resistance loci in maize. Plant J. 106, 526–535. doi: 10.1111/tpj.15183 33533097

[B261] Ye Y.-F. Li Q.-Q. Gang F. Yuan G.-Q. Miao J.-H. Wei L. (2012). Identification of antifungal substance (Iturin A2) produced by *Bacillus subtilis* B47 and its effect on southern corn leaf blight. J. Integr. Agric. 11, 90–99. doi: 10.1016/S1671-2927(12)60786-X

[B262] Yin X. Wang Q. Yang J. Jin D. Wang F. Wang B.. (2003). Fine mapping of the *Ht*2 (*Helminthosporium turcicum* resistance 2) gene in maize. Chin. Sci. Bull. 48, 165–169. doi: 10.1360/03tb9034

[B263] Young N. (1996). QTL mapping and quantitative disease resistance in plants. Annu. Rev. Phytopathol. 34, 479–501. doi: 10.1146/annurev.phyto.34.1.479 15012553

[B264] Yuli D. Lin G. Hongchun R. Niuniu S. Yixin D. Furu C.. (2017). Sensitivity of *Bipolaris maydis* to iprodione and pyraclostrobin and their control efficacy against southern corn leaf blight in Fujian province. Chin. J. Pesticide Sci. 19 (4), 434–440.

[B265] Zaitlin D. DeMars S. Ma Y. (1993). Linkage of rhm, a recessive gene for resistance to southern corn leaf blight, to RFLP marker loci in maize (*Zea mays*) seedlings. Genome 36, 555–564. doi: 10.1139/g93-076 8102347

[B266] Zhan J. Thrall P. H. Burdon J. J. (2014). Achieving sustainable plant disease management through evolutionary principles. Trends Plant Sci. 19, 570–575. doi: 10.1016/j.tplants.2014.04.010 24853471

[B267] Zhang F. (2013). Recognition of corn leaf disease based on quantum neural network and combination characteristic parameter. J. South. Agric. 44 (8), 1286–1290.

[B268] Zhang B. Chi D. Hiebert C. Fetch T. McCallum B. Xue A.. (2019). Pyramiding stem rust resistance genes to race TTKSK (Ug99) in wheat. Can. J. Plant Pathol. 41, 443–449. doi: 10.1080/07060661.2019.1596983

[B269] Zhang X. Qiao Y. Meng F. Fan C. Zhang M. (2018). Identification of maize leaf diseases using improved deep convolutional neural networks. IEEE Access 6, 30370–30377. doi: 10.1109/ACCESS.2018.2844405

[B270] Zhang Y. Xu L. Fan X. Tan J. Chen W. Xu M. (2012). QTL mapping of resistance to gray leaf spot in maize. Theor. Appl. Genet. 125, 1797–1808. doi: 10.1007/s00122-012-1954-z 22903692

[B271] Zhang X. Yang Q. Rucker E. Thomason W. Balint-Kurti P. (2017). Fine mapping of a quantitative resistance gene for gray leaf spot of maize (*Zea mays* L.) derived from teosinte (*Z. mays* ssp. *parviglumis*). Theor. Appl. Genet. 130, 1285–1295. doi: 10.1007/s00122-017-2888-2 28342108

[B272] Zhang Y.-m. Zhang Y. Xie K. (2020). Evaluation of CRISPR/Cas12a-based DNA detection for fast pathogen diagnosis and GMO test in rice. Mol. Breed. 40, 11. doi: 10.1007/s11032-019-1092-2

[B273] Zhao H. Gao Z.-g. Zhang X.-f. Zhuang J.-h. Sui H. (2008). Population of physiological races of *Setosphaeria turcica* and its dynamic analysis in China. J. Shenyang Agric. Univ. 39 (5), 551–555.

[B274] Zhao J. Jiang X. Jia H. Li S. Shi J. Zhang H. (2012). Identification and evaluation of physiological races of *Bipolaris maydis* in Huanghuaihai region. J. Hebei Agric. Sci. 16, 47–49.

[B275] Zhu X. Reid L. Woldemariam T. (2011). Pathogenic races of *Exserohilum turcicu*m on corn in Ontario and Quebec. Phytopathology 101 (6), S252.

[B276] Zhu X. Reid L. Woldemariam T. Tenuta A. Schaafsma A. (2002). First report of gray leaf spot caused by *Cercospora zeae-maydis* on corn in Ontario, Canada. Plant Dis. 86, 327–327. doi: 10.1094/PDIS.2002.86.3.327C 30818620

[B277] Zwonitzer J. C. Bubeck D. M. Bhattramakki D. Goodman M. M. Arellano C. Balint-Kurti P. J. (2009). Use of selection with recurrent backcrossing and QTL mapping to identify loci contributing to southern leaf blight resistance in a highly resistant maize line. Theor. Appl. Genet. 118, 911–925. doi: 10.1007/s00122-008-0949-2 19130030

